# Brain tissue heterotopic in the adrenal gland in a child: a scarce case report

**DOI:** 10.1186/s12887-023-04478-0

**Published:** 2024-02-03

**Authors:** Chenghao Zhanghuang, Chengchuang Wu, Junling Chen, Fengming Ji, Zhigang Yao, Li Li, Zhen Yang, Haoyu Tang, Kun Zhang, Yu Hang, Yucheng Xie, Bing Yan

**Affiliations:** 1 Department of Urology, Kunming Children’s Hospital (Children’s Hospital affiliated to Kunming Medical University), Kunming, PR China Yunnan Province Clinical Research Center for Children’s Health and Disease, 288 Qianxing Road, Kunming, 650228 Yunnan China; 2 Yunnan Clinical Medical Center for Pediatric Diseases; Yunnan Key Laboratory of Children’s Major Disease Research, Kunming Children’s Hospital (Children’s Hospital affiliated to Kunming Medical University), Kunming, People’s Republic of China; 3 Laboratory Medicine Department, Kunming Children’s Hospital (Children’s Hospital affiliated to Kunming Medical University), Kunming, People’s Republic of China; 4 Department of Oncology; Kunming Children’s Solid Tumor Diagnosis and Treatment Center, Kunming Children’s Hospital (Children’s Hospital affiliated to Kunming Medical University), Kunming, People’s Republic of China; 5 Department of Pathology, Kunming Children’s Hospital (Children’s Hospital affiliated to Kunming Medical University), Kunming, People’s Republic of China

**Keywords:** Choristoma, Adrenal gland neoplasm, Adrenal glands, Diagnosis, Clinical pathology, Treatment

## Abstract

Heterotopic brain tissue is rare and has not been reported. Our center made the first report. 4 years and 2 months old Girl presented with a cystic mass in the right adrenal gland 2 weeks after right upper abdominal pain. The operation was successful, and the diagnosis was confirmed by postoperative pathology. 6 months after the procedure, the incision healed well without recurrence. This case report has a detailed diagnosis and treatment process and satisfactory examination results. It can provide a reference for diagnosing and treating clinical HBT and reduce the risk of misdiagnosis and mistreatment.

**Supplementary Information:**

The online version contains supplementary material available at 10.1186/s12887-023-04478-0.

## Background

Heterotopic brain tissue (HBT) is a rare clinical lesion that is extremely difficult to diagnose before surgery. Currently, the main reports focus on the nasal cavity and soft palate [1], and the ectopic brain tissue in the nasal cavity is called nasal glioma [2]. It is rare to report that HBT can be found in neck and lung tissue [3–5]. At present, no HBT from the adrenal gland has been reported. A case of adrenal HBT in children admitted to our center is declared as follows.

## Case report

**Fig. 1:**
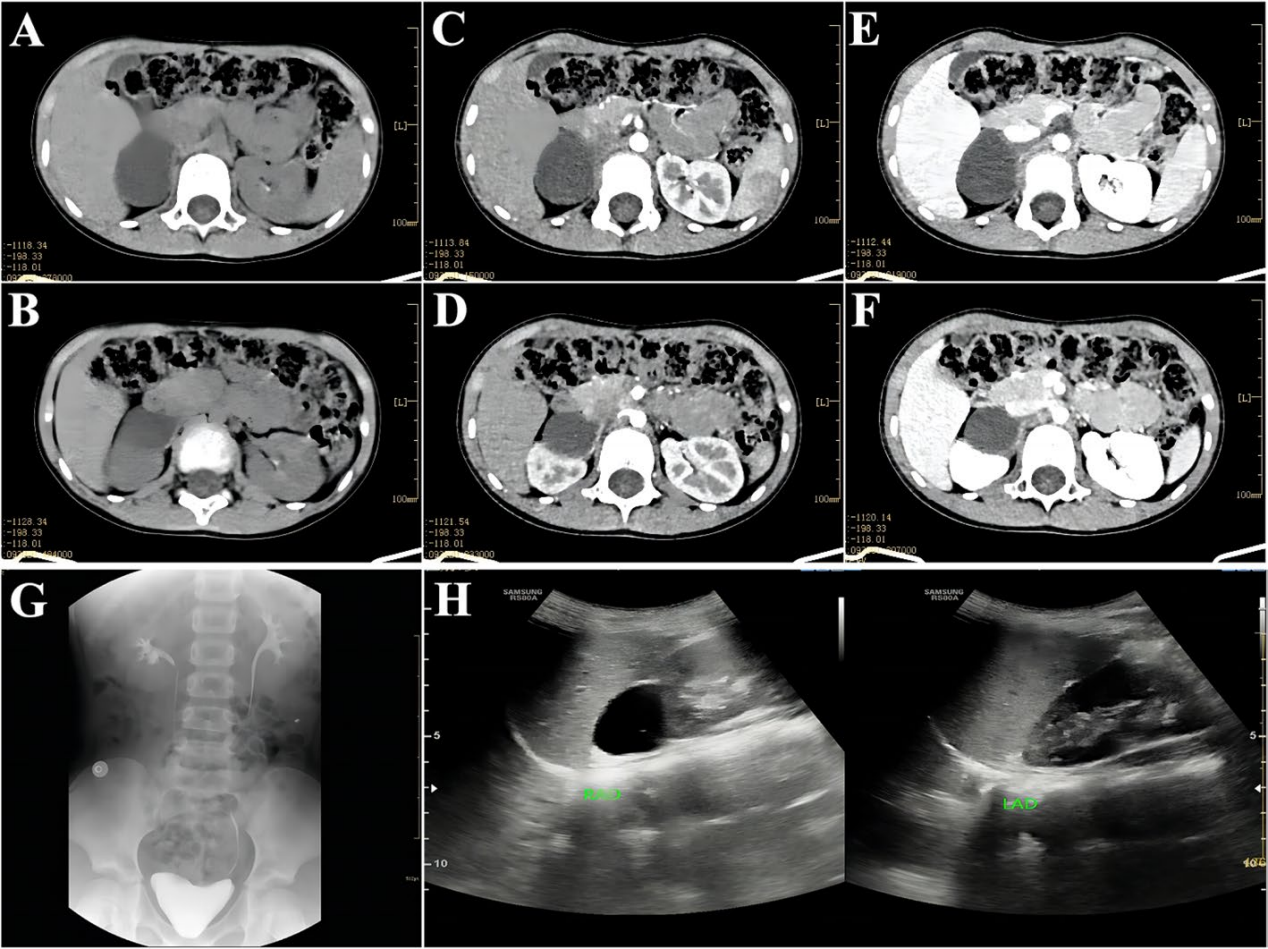
Preoperative imaging findings; **A**-**B**: adrenal CT scan findings; **C**-**D**: findings in the arterial phase of adrenal enhanced CT; **E**-**F**: findings in the venous phase of adrenal enhanced CT; **G**: findings of intravenous urography (IVP); **H**: Findings on color Doppler ultrasound of the adrenal gland

The patient was a girl aged 4 years and 2 months. She was admitted to the hospital due to “A cystic mass was found in the right adrenal gland for two weeks.”. Two weeks ago, the child developed severe right upper abdominal pain without an apparent cause, accompanied by nausea and vomiting twice, and the vomit was content. Color Doppler ultrasound (US) and computed tomography (CT) plain scan in the local hospital revealed a cystic mass in the right adrenal gland, about 4.3*2.8 cm in size. There was no urinary pain, dysuria, fever, abdominal distension, diarrhea, edema, frequent urination, or urgency during the disease. She went to our hospital for further treatment and was admitted with a right adrenal mass of unknown etiology. The patient had a regular diet and slept in the medical history; no abnormalities in the bowel or urine were found. After admission, relevant examinations were performed. CT plain and enhanced scans showed an oval low-density lesion in the right adrenal gland area, with a CT value of about 14HU, a clear edge, and a size of about 3.7 cm × 3.3 cm × 3.3 cm, and no enhancement was observed. It was reported as an oval hypodense lesion in the right adrenal region, with a possible cyst (Fig. 1A-F). Intravenous pyelography (IVP) revealed a downward and lateral displacement of the right kidney, and imaging suggested that the mass originated from the upper pole of the right kidney or the adrenal gland (Fig. 1G). US of the urinary system showed a well-defined oval shape with anechoic area and flocculent hypoecho. On ultrasound, a cystic-solid mass in the right middle abdomen adjacent to the upper pole of the right kidney was considered. It was considered a right renal cyst (Fig. 1H). Laboratory tests showed that urinary vanillylmandelic acid (VMA) was 2.71 mg/24H, metanephrine was 0.13 nmol/L, normetanephrine was 0.39 nmol/L, and other adrenal function indexes were normal.

**Fig. 2:**
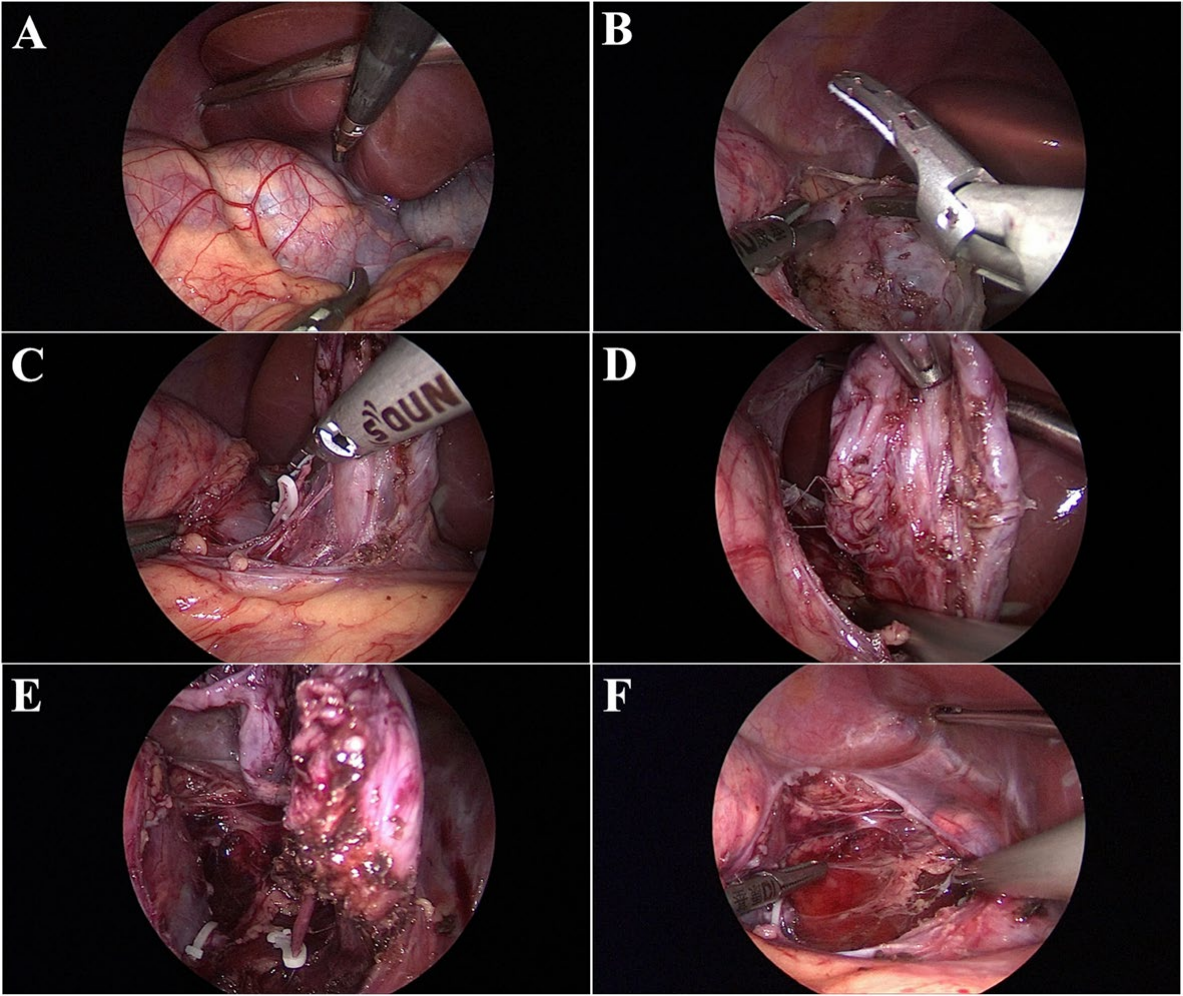
Intraoperative findings of laparoscopic surgery; **A** The mass was located in the right adrenal gland under laparoscopy. **B** the upper cyst wall of the mass was carefully dissociated; **C** the branch vessels were clamped with a biological hemostatic clamp; **D** the cyst wall of the lower part of the mass was carefully dissociated; **E** The adhesion of the mass to the upper right kidney was clamped with a bio-hemostatic clamp. **F** Surgical view after removal of the mass

After improvement of relevant preoperative examination, no surgical contraindication was found, and laparoscopic right adrenal mass resection was performed under general anesthesia. An abdominal incision opened the retroperitoneal space during the operation, and the right adrenal cystic mass was found (Fig. 2A). The size of the group was about 4 cm*5 cm*5 cm, which was cystic. There was no apparent boundary between the mass and the adrenal gland and a fibrous cord-like connection with the upper kidney (Fig. 2B-D). After the biological clip clipped the fibrous cord-like link, the cyst and normal adrenal tissue were separated by an ultrasonic scalpel. Cyst tissue was dissected thoroughly, and excised tissue was removed for pathological examination (Fig. 2E). The procedure was uneventful, and no bleeding was seen in the surgical field after resection (Fig. 2F). The wound healed well after the dressing change 2 days after surgery, and the patient was discharged 5 days after surgery.

**Fig. 3:**
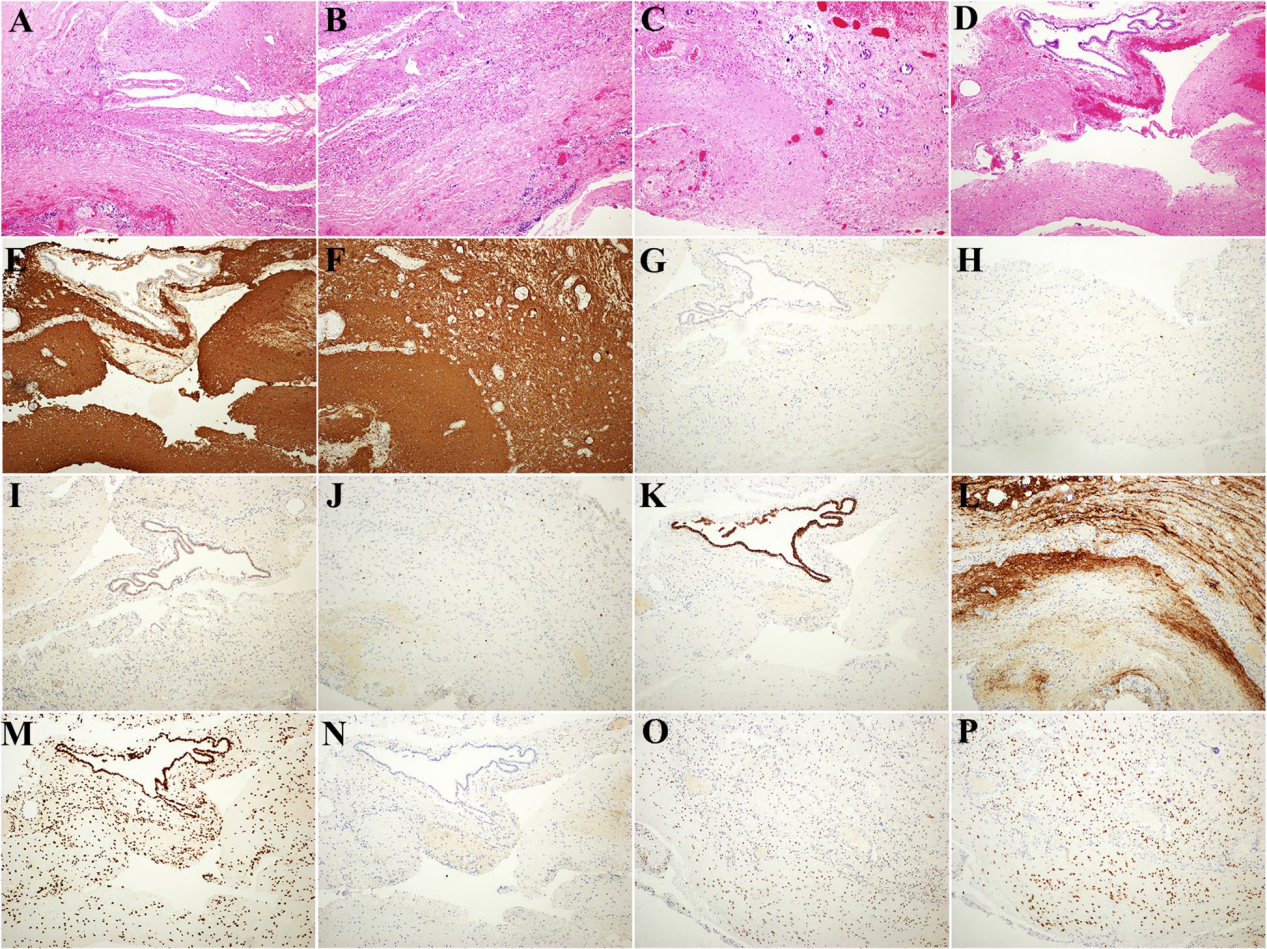
Postoperative pathological examination and immunohistochemical results: **A**-**B** the wall of the mass cyst was lined with glia, and A small amount of adrenal cortex was seen in the lower left corner (HE), 10X). **C** Full of glial cells in the upper left, with spammed calcifications shown above (HE, 10X); **D** Localized ependymal epithelium (HE, 4X) lining the wall of the mass capsule. **E**-**P** Postoperative immunohistochemical findings (E-F: GFAP, G-H: Ki-67, I: CK20, J: Olig, K: CK7, L: Syn, M: H3K27me3, N: H3K27M, O: ATRX, P: SOX2) (E-P: 10X)

Postoperative pathological examination showed that the cyst wall was lined with glial components, fibrous tissue and small blood vessel proliferation, scattered chronic inflammatory cells, a small amount of calcification, multinucleated giant cells, and a lumen covered with cuboidal epithelium (Fig. 3A-B). Numerous nerve fibers and ganglion cells were found locally around the wall, and stellate adrenal tissue was seen (Fig. 3C-D). Immunohistochemical results: CK7 (focally +), Syn (+), CK20 (weakly +), Olig2 (scattered +), GFAP (+), Ki-67 (+), ATRX (+), SOX2 (+), H3K27M (−), H3K27me3 (+) (Fig. 3E-P).

The microscopic and immunohistochemical results diagnosed the patient with heterotopic brain tissue. The patient was reexamined 6 months after surgery, and no recurrence or metastasis was found in the asymptomatic survival.

## Discussion

Human tissue heterotopia refers to human tissue growth in a part of the body other than that organ. Tumors arising in ectopic tissues were considered primary tumors. HBT is rare clinically, and the few existing reports mainly focus on the nasal cavity and soft palate. It is worth noting that all HBT reported at present originated from the respiratory tract. Although most HBT are benign tumors, they are often accompanied by some respiratory compression symptoms, leading to respiratory obstruction in children and requiring urgent intervention [6]. To our knowledge, this is the first reported case so far.

The adrenal glands are paired endocrine glands that are soft and pale yellow, and the right adrenal gland is triangular. The medial part of the anterior part had no peritoneum and was in direct contact with the inferior vena cava. The lateral part was contiguous with the liver. The posterior of the gland is slightly convex to the diaphragm; The bottom depression, called the renal surface, lies tightly on the upper end of the right kidney, and its medial edge is adjacent to the celiac ganglion, with abundant blood supply [7]. The adrenal gland is divided into two parts, the cortex and the medulla, which have entirely different embryonic origins, the former from the mesoderm and the latter from the ectoderm. However, gliomas mainly originate from the ectoderm, so the adrenal medulla is considered the most likely source. Although HBT is mostly benign if the cyst produces compression symptoms in the adrenal gland, it may cause clinical signs such as hypertension and abdominal pain [8]. The adrenal gland should be considered for retroperitoneal cystic lesions in the upper abdomen. US is the first choice of examination method, which is economical, painless and safe, and can significantly improve the diagnostic rate. Contrast-enhanced CT is a more accurate examination method for diagnosing adrenal cystic lesions [9]. In this case, preoperative examination showed that the cystic lesion originated from the adrenal gland, but the nature was unknown.

The preoperative diagnosis of HBT is challenging, and the diagnosis is mainly based on postoperative pathological examination and immunohistochemical results. The differential diagnosis of teratoma especially needs to be differentiated from mature teratoma. Teratoma is generally a single intact cystic mass with relatively clear boundaries with surrounding tissues, and the cyst contains hair and fatty structures. In addition, the histological components of teratoma showed endodermal, mesodermal and ectodermal components in different proportions [10]. In this case, hematoxylin-eosin (HE) staining showed only ectodermal and glial cell components. The cyst wall was lined with neuroglia and ependymal epithelium, and a small amount of adrenal cortex could be seen, which could be identified. This study confirmed that the mass was derived from the adrenal gland by the results of multiple immunohistochemistry and confirmed that the child’s cystic mass was diagnosed as HBT.

HBT has a good prognosis. Complete surgical resection of the HBT mass is curative, but incomplete resection may cause recurrence [4]. Therefore, careful dissection of the cyst wall during the operation and regular postoperative review are critical. In this case, the mass was dissected entirely along the cyst wall during the process, and B-ultrasound was performed at 1, 3 and 6 months after the operation. The prognosis was good, and the child did not have abdominal pain again.

## Conclusion

HBT originating from adrenal glands has not been reported. Attention should be paid if there is an unexplained adrenal cyst with hypertension, abdominal pain, and other clinical manifestations. HBT must be differentiated from mature teratoma and depends on postoperative pathological examination for diagnosis. The prognosis of HBT is good. Careful dissection of the cyst wall during the operation and regular postoperative review are the keys to cure.

### Supplementary Information

**Additional file 1.**

## Data Availability

The datasets used during the current study available from the corresponding author on reasonable request.
