# Supplementary material for: Brain tissue heterotopic in the adrenal gland in a child: a scarce case report
Source: BMC Pediatr. 2024 Feb 3;24:97. doi: 10.1186/s12887-023-04478-0 (PMC10837895; doi:10.1186/s12887-023-04478-0)

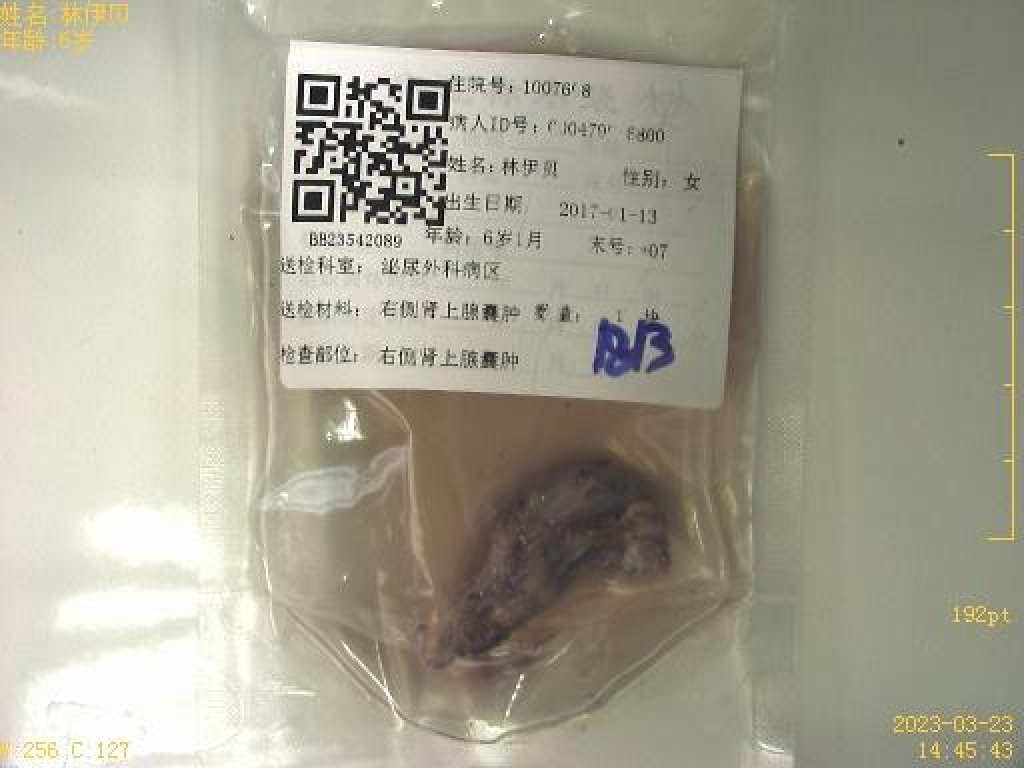

姓名：林伊贝
性别：女
BB23542089
年龄：6岁1月
送检科室：泌尿外科病区
送检材料：右侧肾上腺囊肿
检查部位：右侧肾上腺囊肿
192pt
2023-03-23
14 45 43

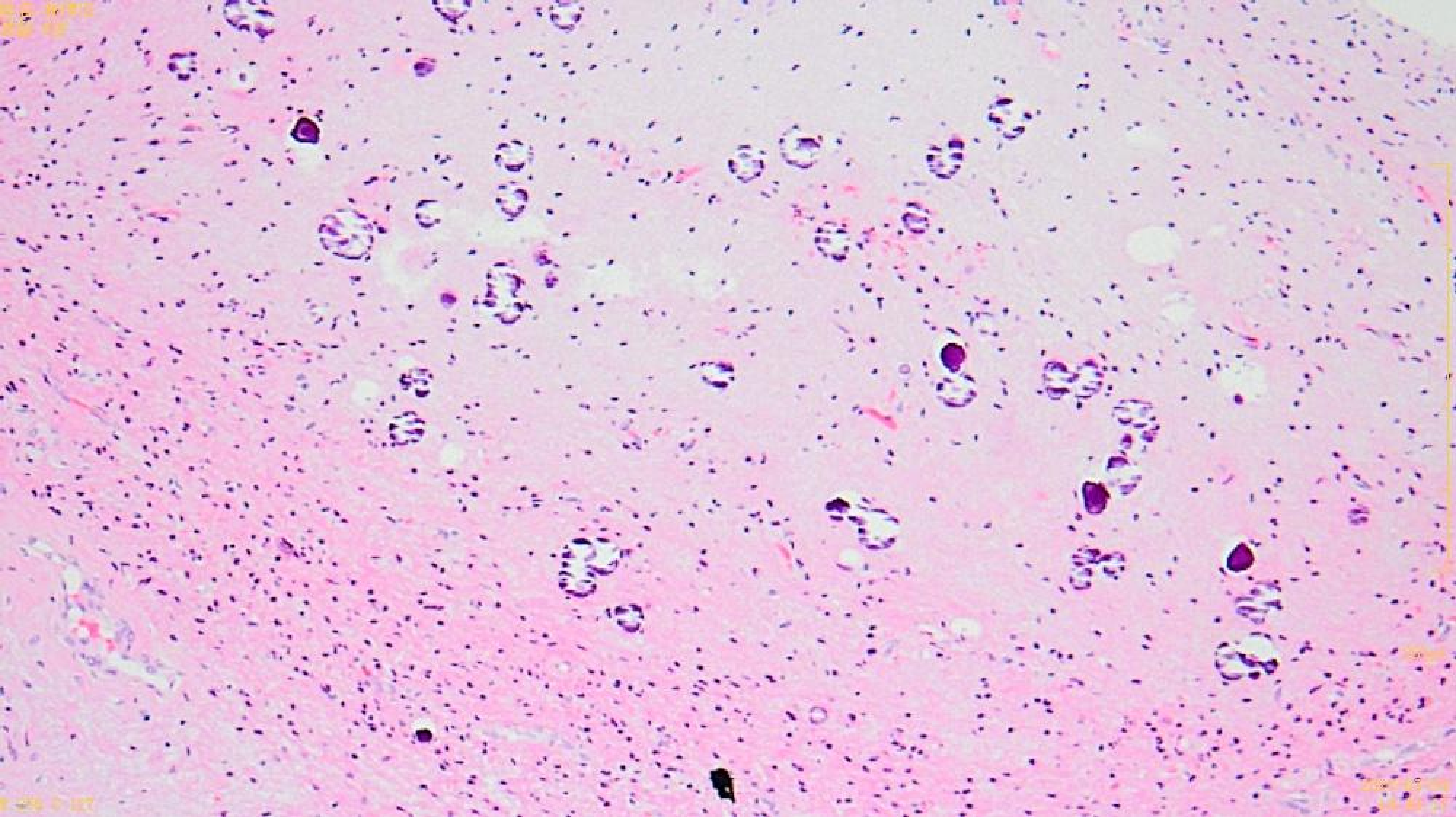

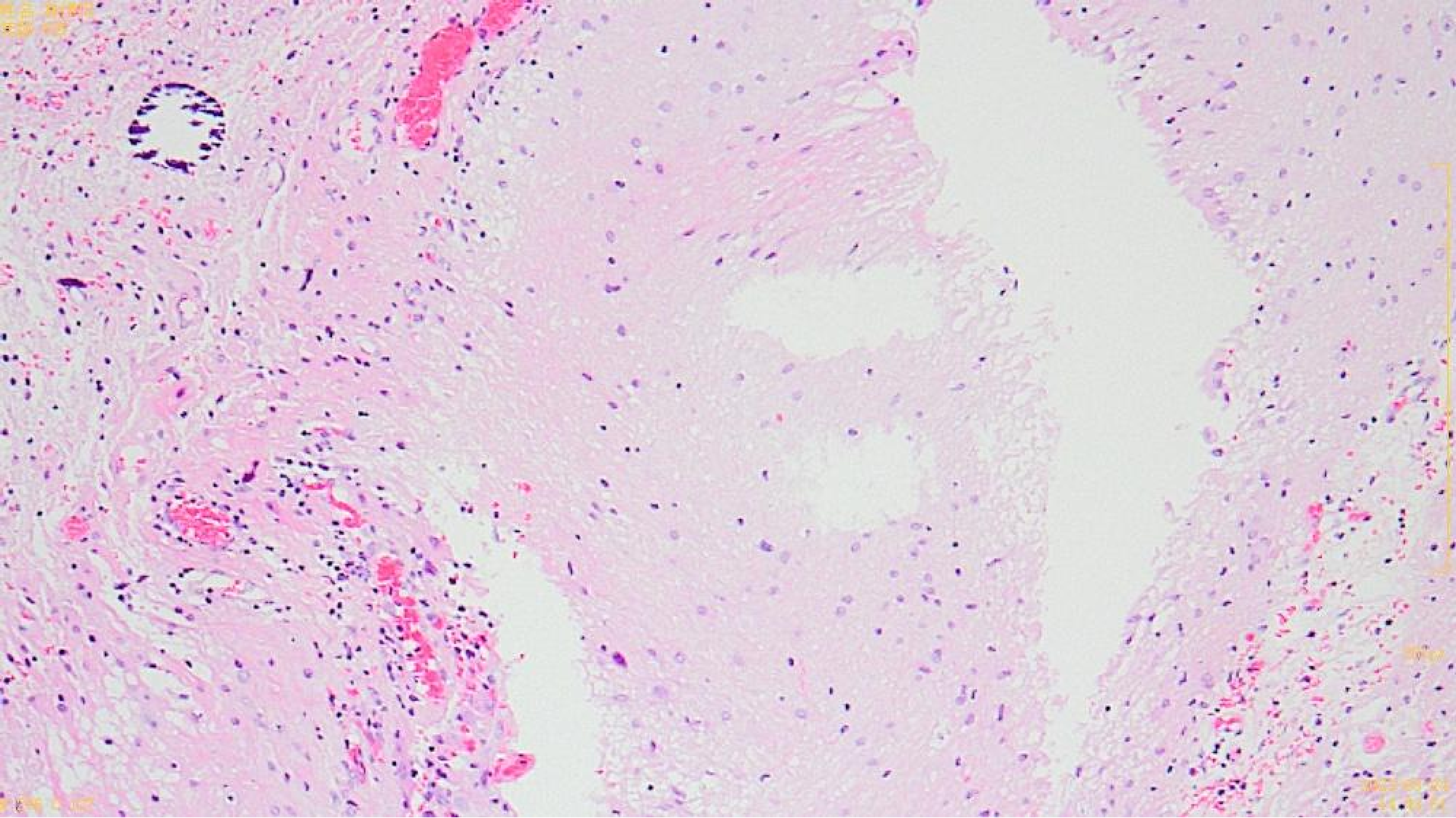

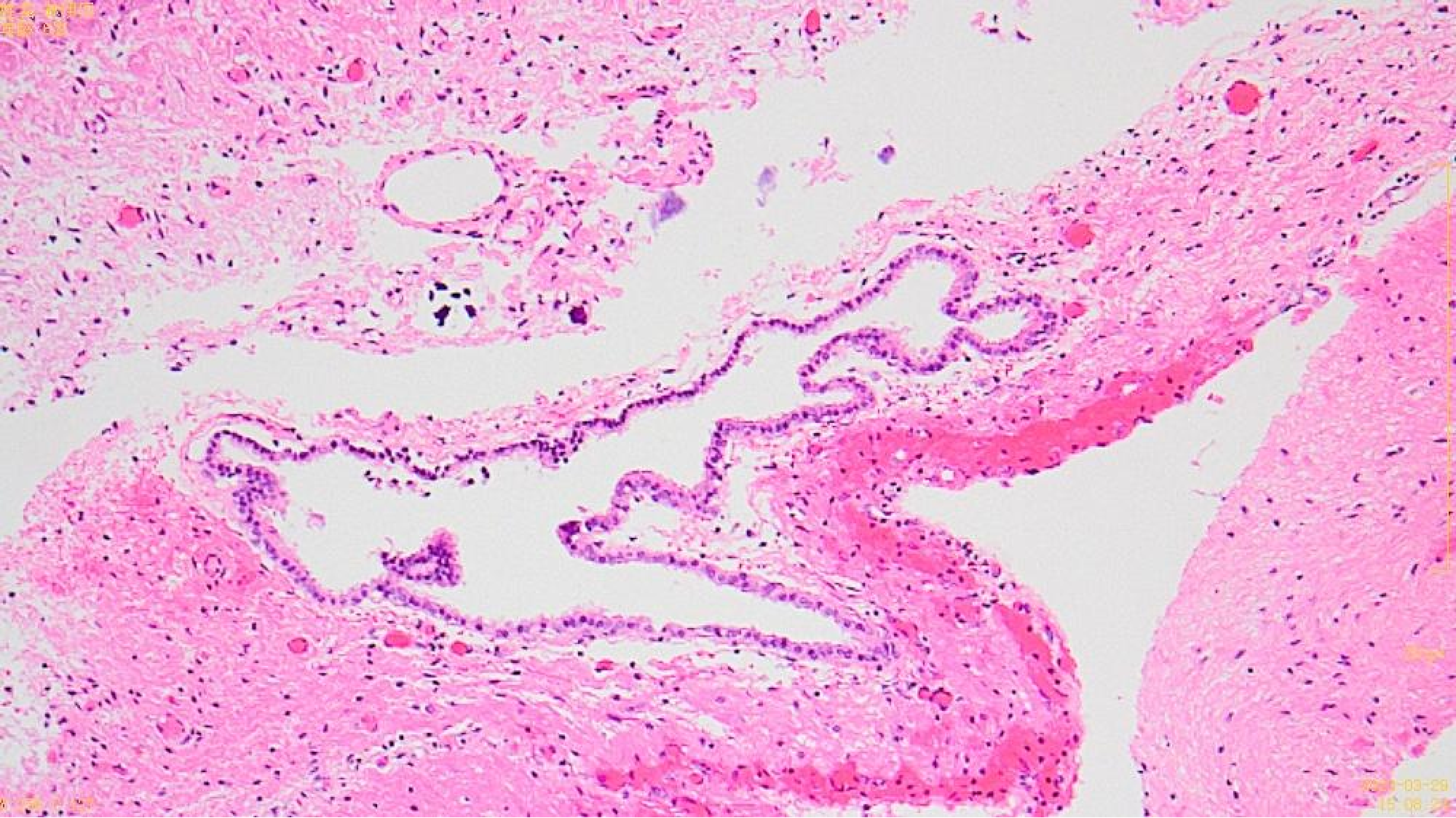

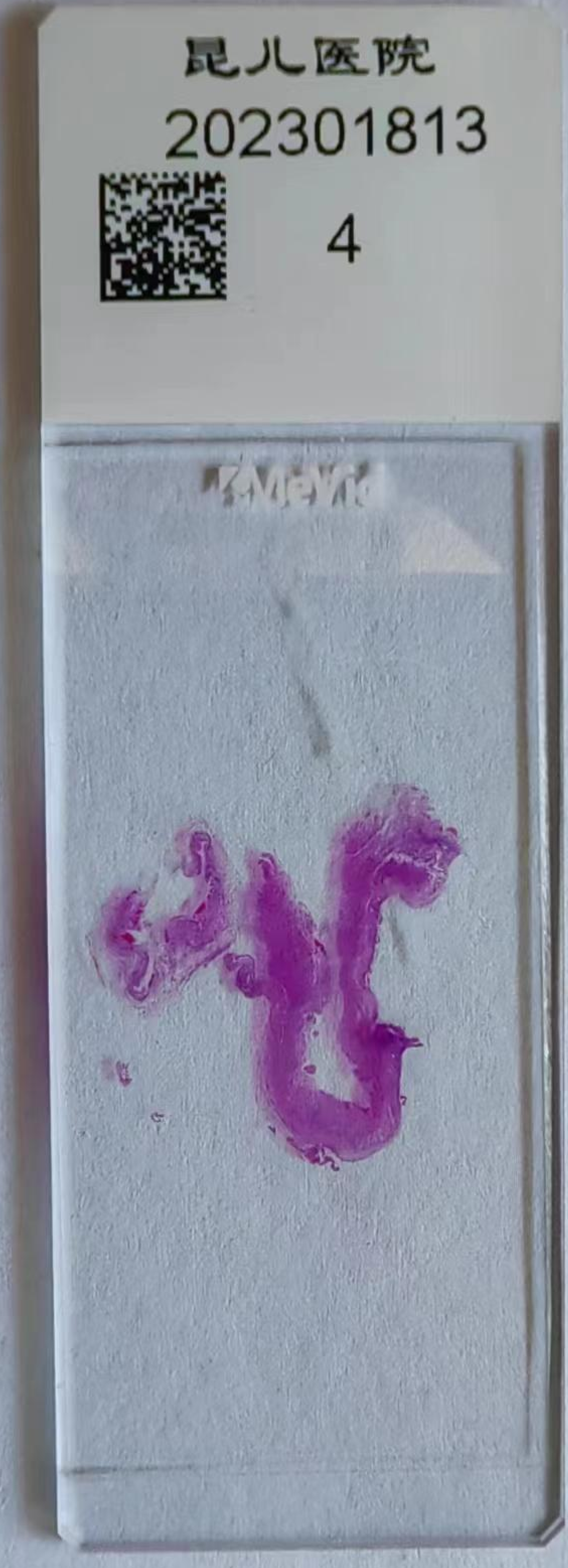
昆儿医院
202301813
4

姓名:林伊贝
编号:CT343419
年龄:6岁
性别:女
部位:腹部

序列:6
序号:19
标号:200
矩阵:512x512

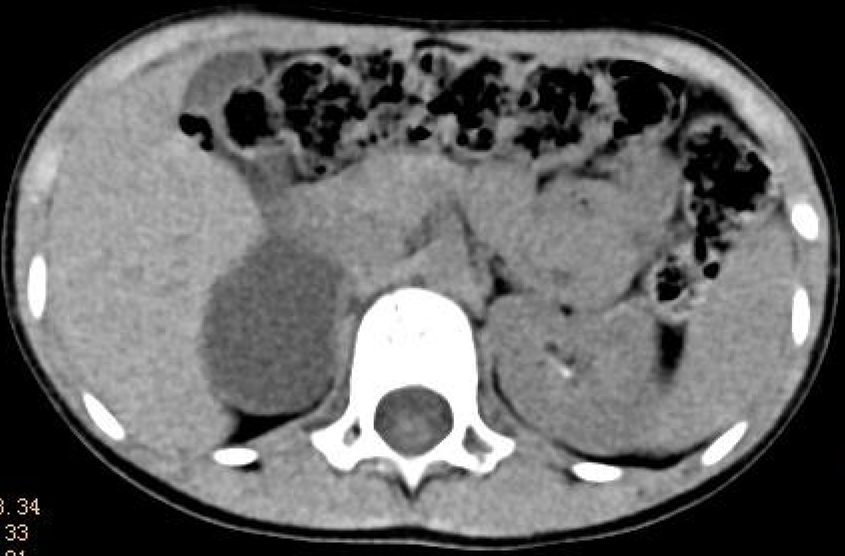

HF:-1118.34
AP:-198.33
RL:-118.01
A:09[illegible]78000
120 kV
103 mA
2023-03-13
09:30:28
W:200 C:40

层厚:5.0
位置:-1118.3
体位:HFS
视野:221x221

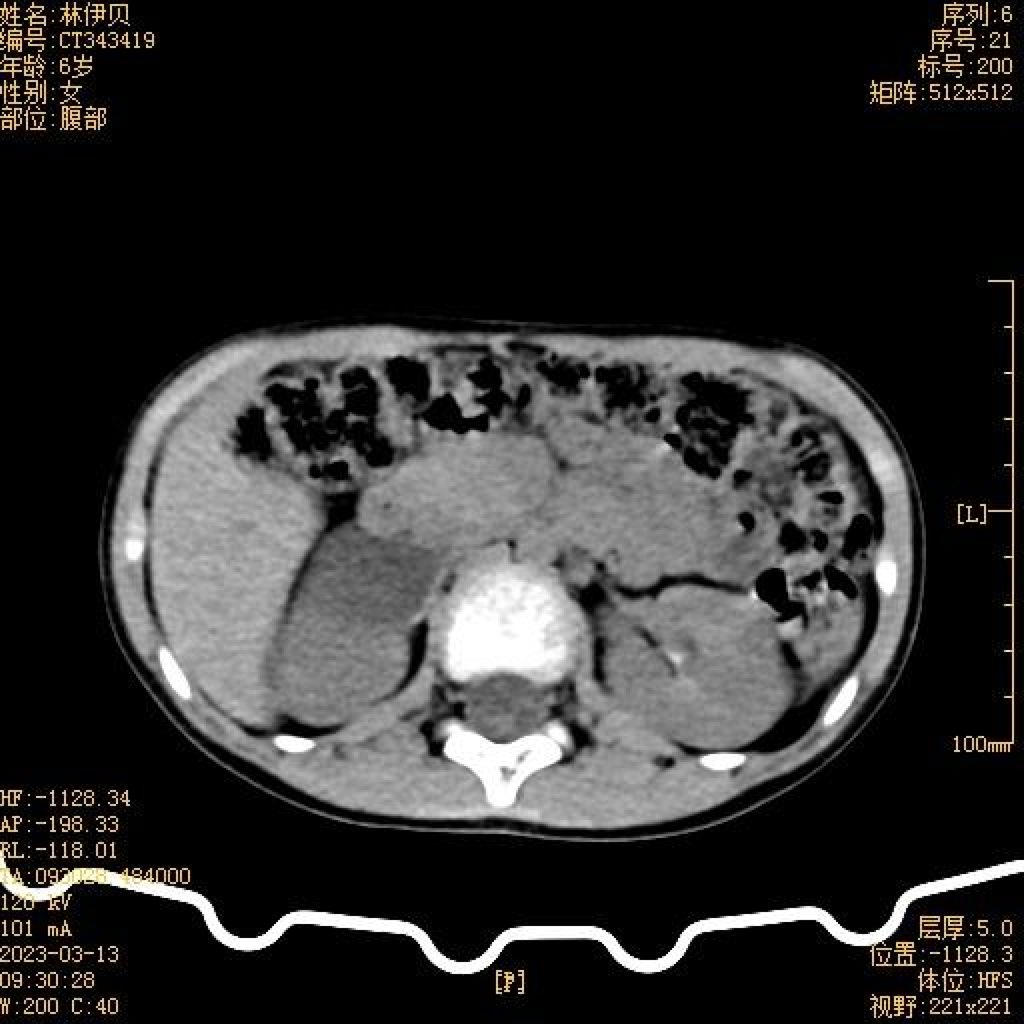
姓名:林伊贝
编号:CT343419
年龄:6岁
性别:女
部位:腹部
序列:6
序号:21
标号:200
矩阵:512x512
[L]
100mm
HF:-1128.34
AP:-198.33
RL:-118.01
120 kV
101 mA
2023-03-13
09:30:28
W:200 C:40
[P]
层厚:5.0
位置:-1128.3
体位:HFS
视野:221x221

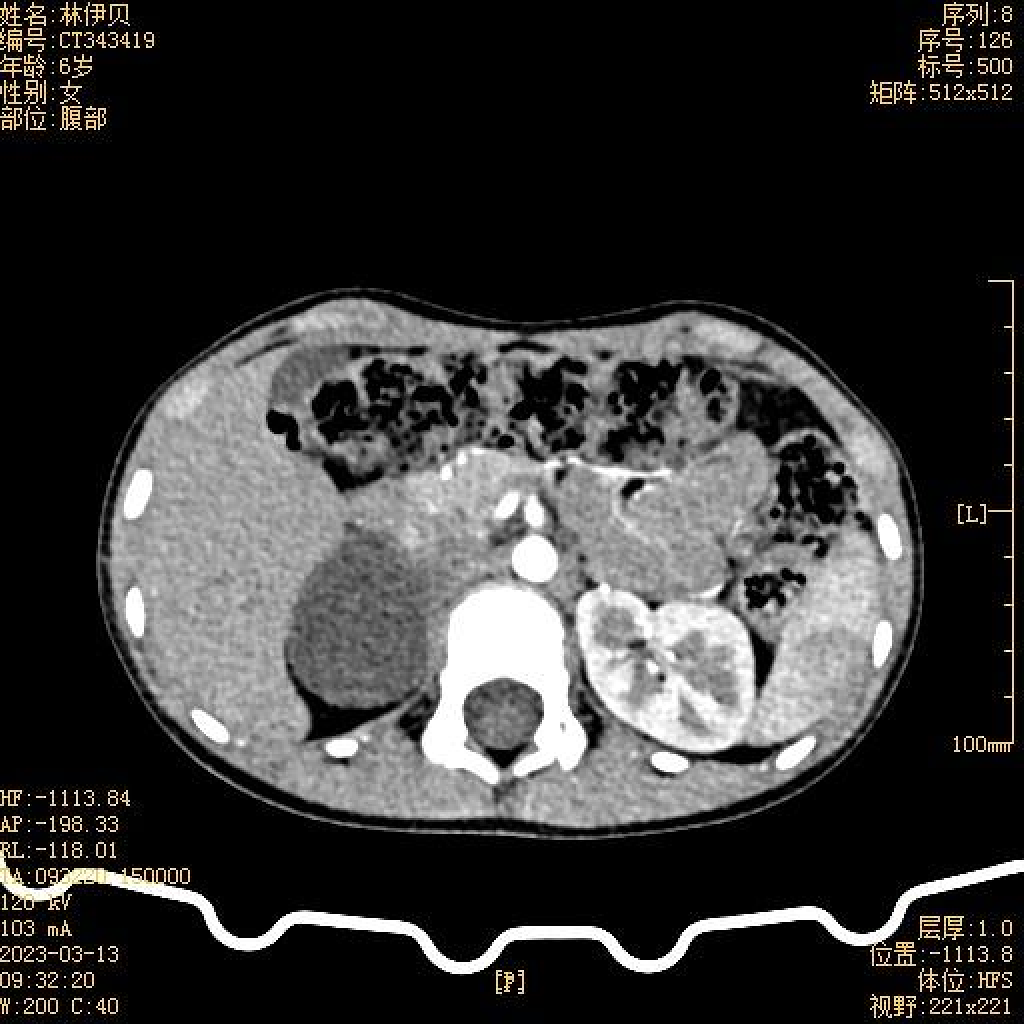
姓名:林伊贝
编号:CT343419
年龄:6岁
性别:女
部位:腹部
序列:8
序号:126
标号:500
矩阵:512x512
[L]
100mm
HF:-1113.84
AP:-198.33
RL:-118.01
150000
120 kV
103 mA
2023-03-13
09:32:20
W:200 C:40
[P]
层厚:1.0
位置:-1113.8
体位:HFS
视野:221x221

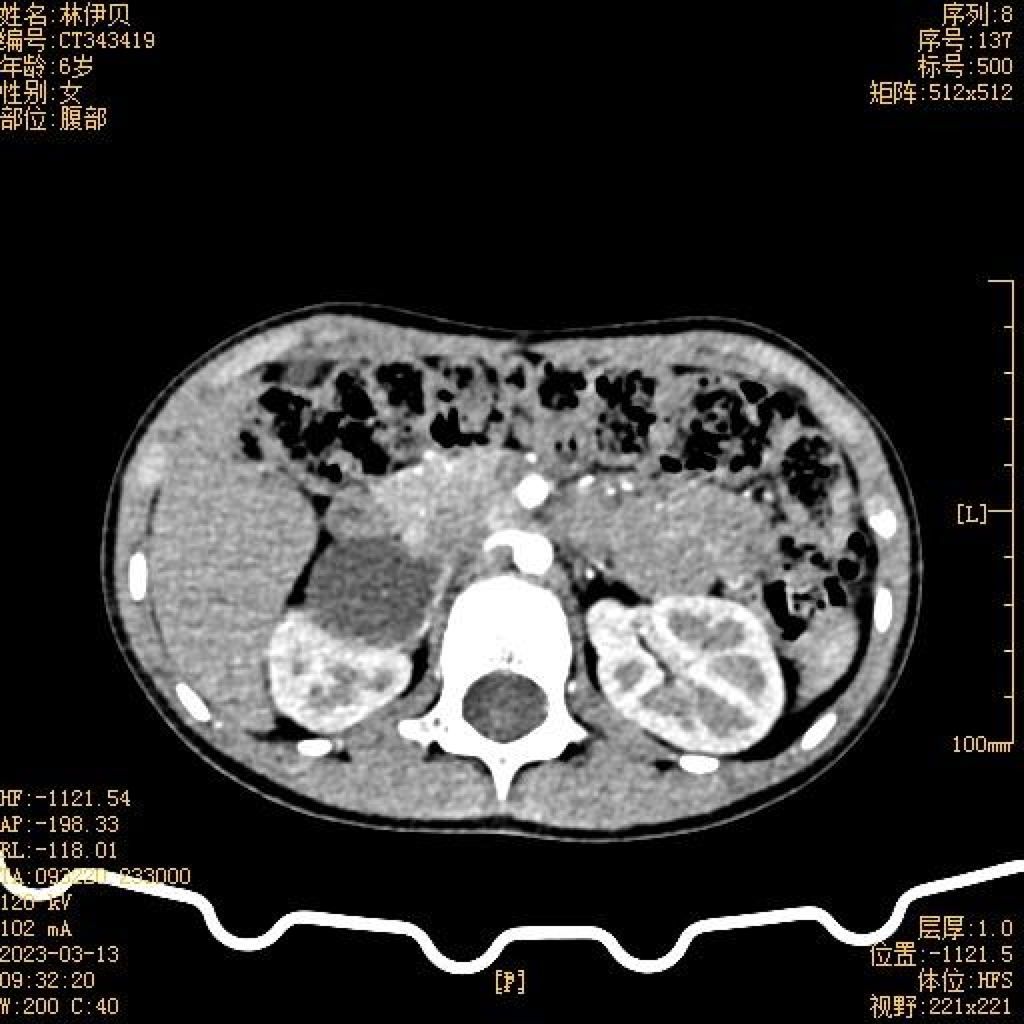

姓名:林伊贝
编号:CT343419
年龄:6岁
性别:女
部位:腹部
序列:8
序号:137
标号:500
矩阵:512x512
[L]
100mm
HF:-1121.54
AP:-198.33
RL:-118.01
A:09
33000
120 kV
102 mA
2023-03-13
09:32:20
W:200 C:40
[P]
层厚:1.0
位置:-1121.5
体位:HFS
视野:221x221

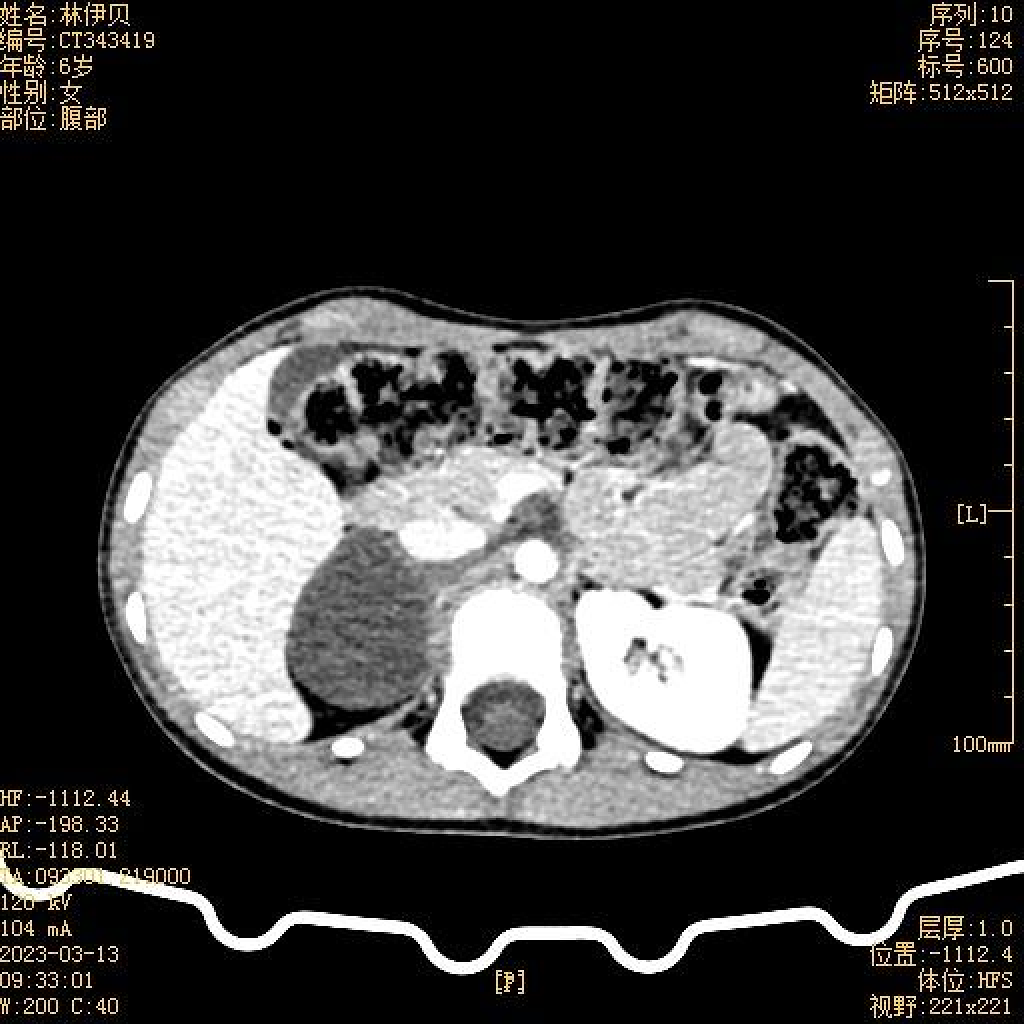
姓名:林伊贝
编号:CT343419
年龄:6岁
性别:女
部位:腹部
序列:10
序号:124
标号:600
矩阵:512x512
[L]
100mm
HF:-1112.44
AP:-198.33
RL:-118.01
120 kV
104 mA
2023-03-13
09:33:01
W:200 C:40
[P]
层厚:1.0
位置:-1112.4
体位:HFS
视野:221x221

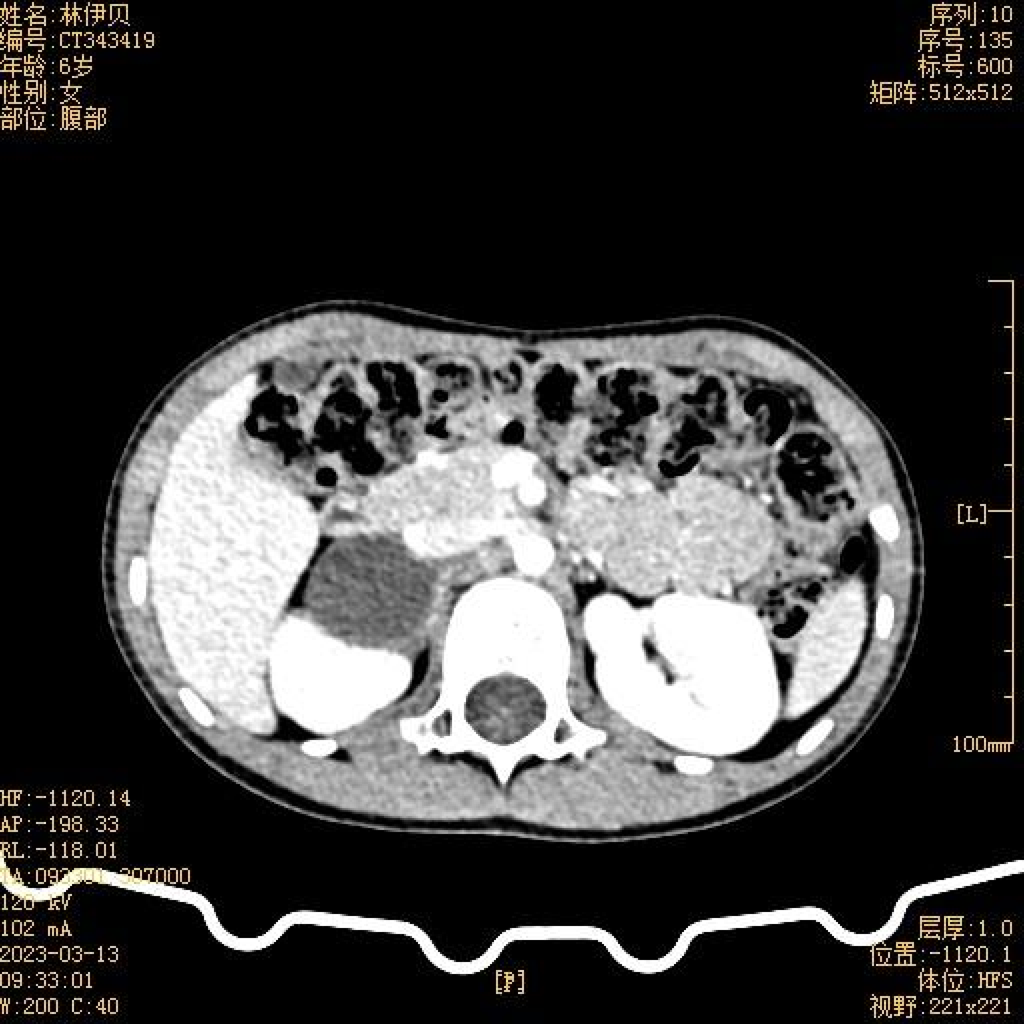
姓名:林伊贝
编号:CT343419
年龄:6岁
性别:女
部位:腹部
序列:10
序号:135
标号:600
矩阵:512x512
[L]
100mm
HF:-1120.14
AP:-198.33
RL:-118.01
120 kV
102 mA
2023-03-13
09:33:01
W:200 C:40
[P]
层厚:1.0
位置:-1120.1
体位:HFS
视野:221x221

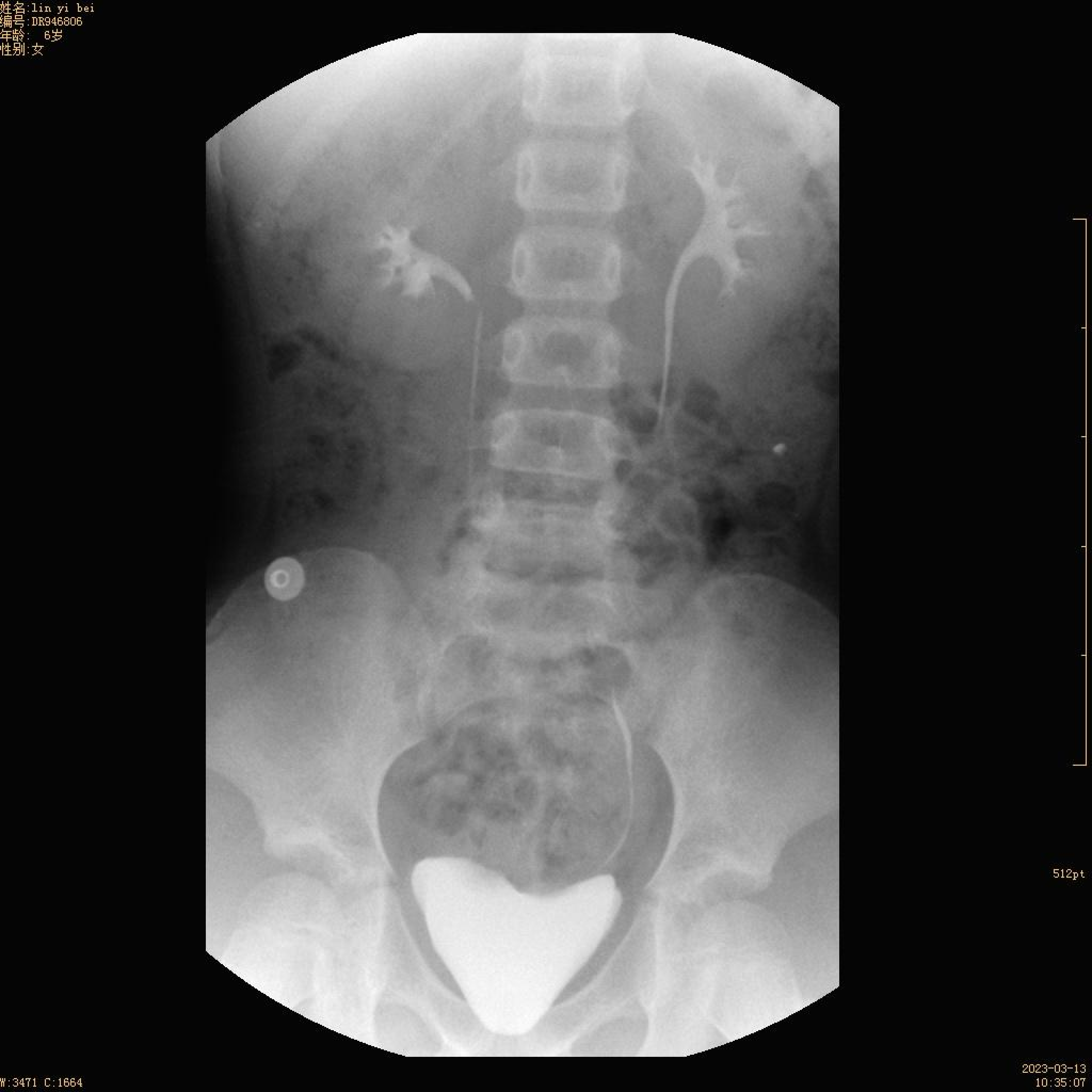
姓名:lin yi bei
编号:DR946806
年龄: 6岁
性别:女
512pt
W:3471 C:1664
2023-03-13
10:35:07

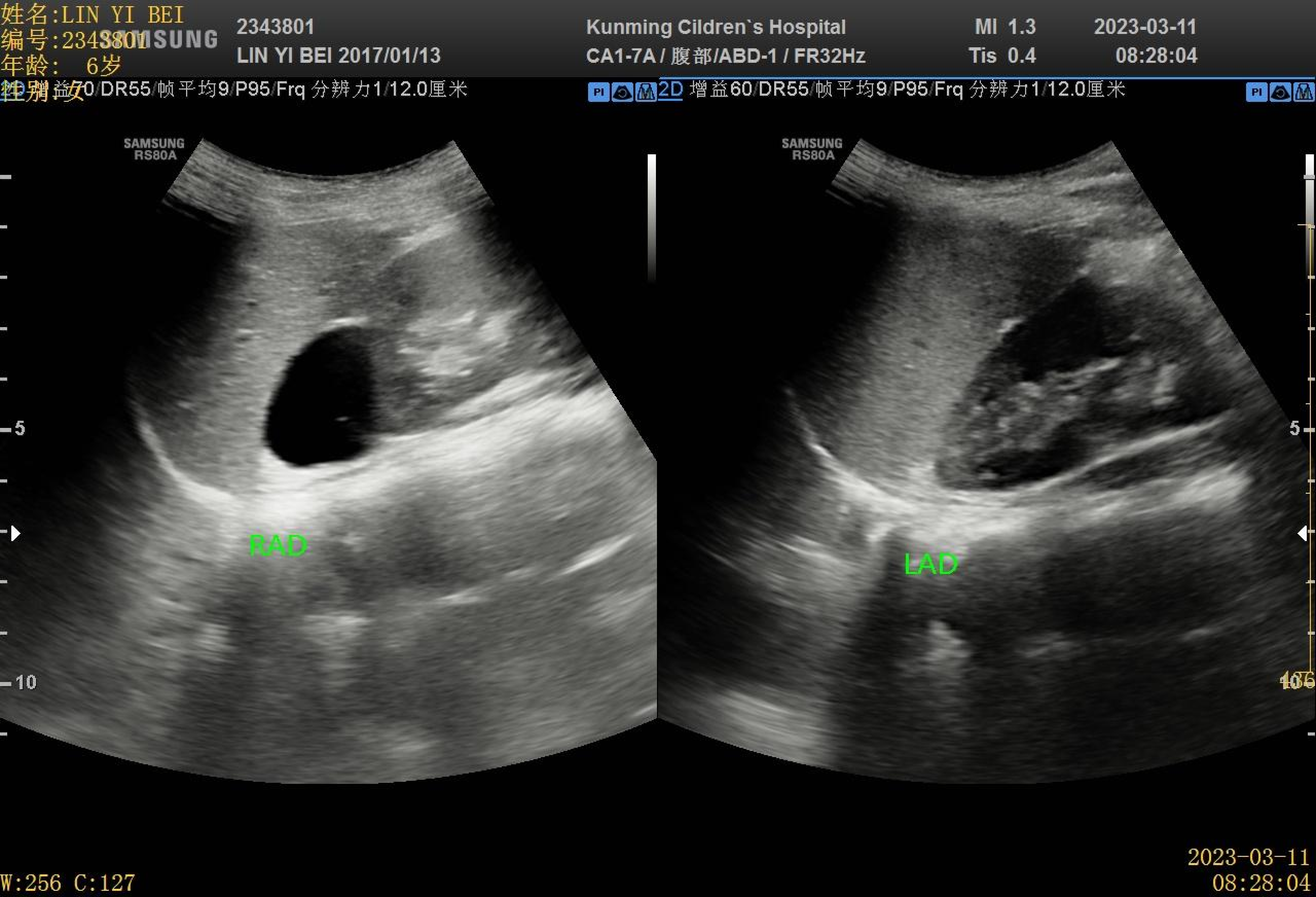

姓名:LIN YI BEI
编号:2343801
年龄: 6岁
2343801
LIN YI BEI 2017/01/13
Kunming Cildren`s Hospital
CA1-7A / 腹部/ABD-1 / FR32Hz
MI 1.3
Tis 0.4
2023-03-11
08:28:04
/DR55/帧平均9/P95/Frq 分辨力1/12.0厘米
2D 增益60/DR55/帧平均9/P95/Frq 分辨力1/12.0厘米
SAMSUNG RS80A
SAMSUNG RS80A
RAD
LAD
5
10
5
2023-03-11
08:28:04
W:256 C:127

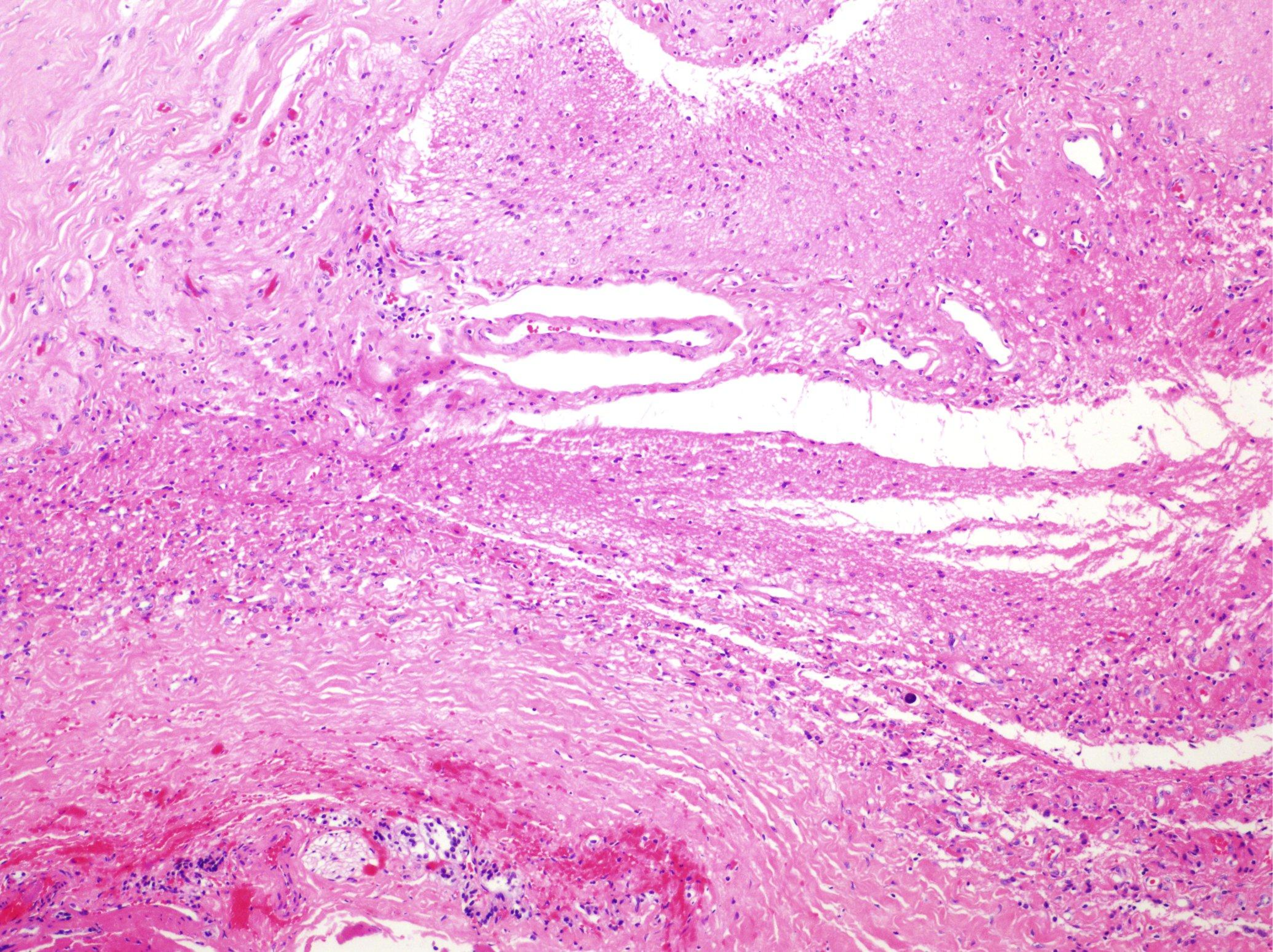

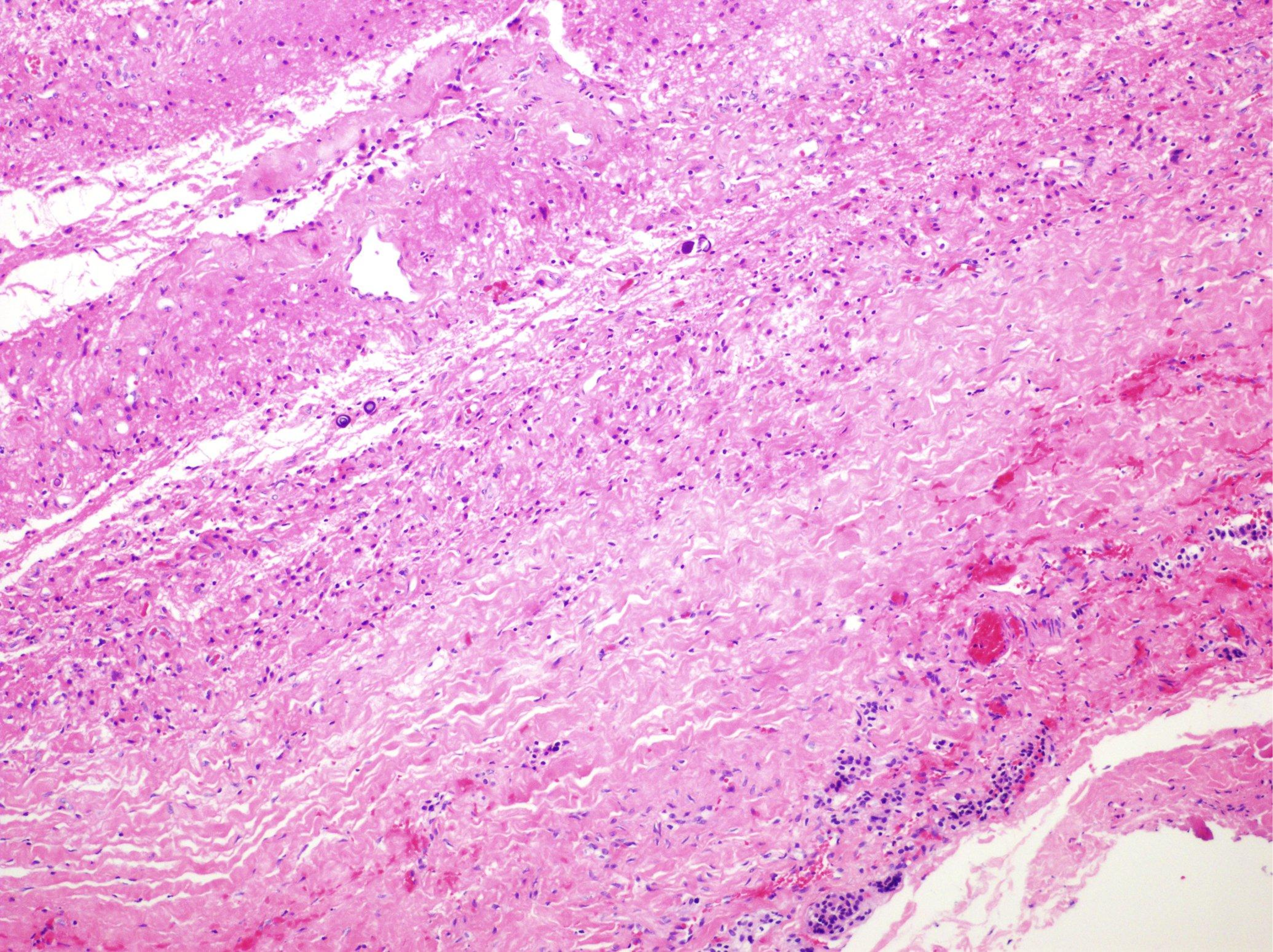

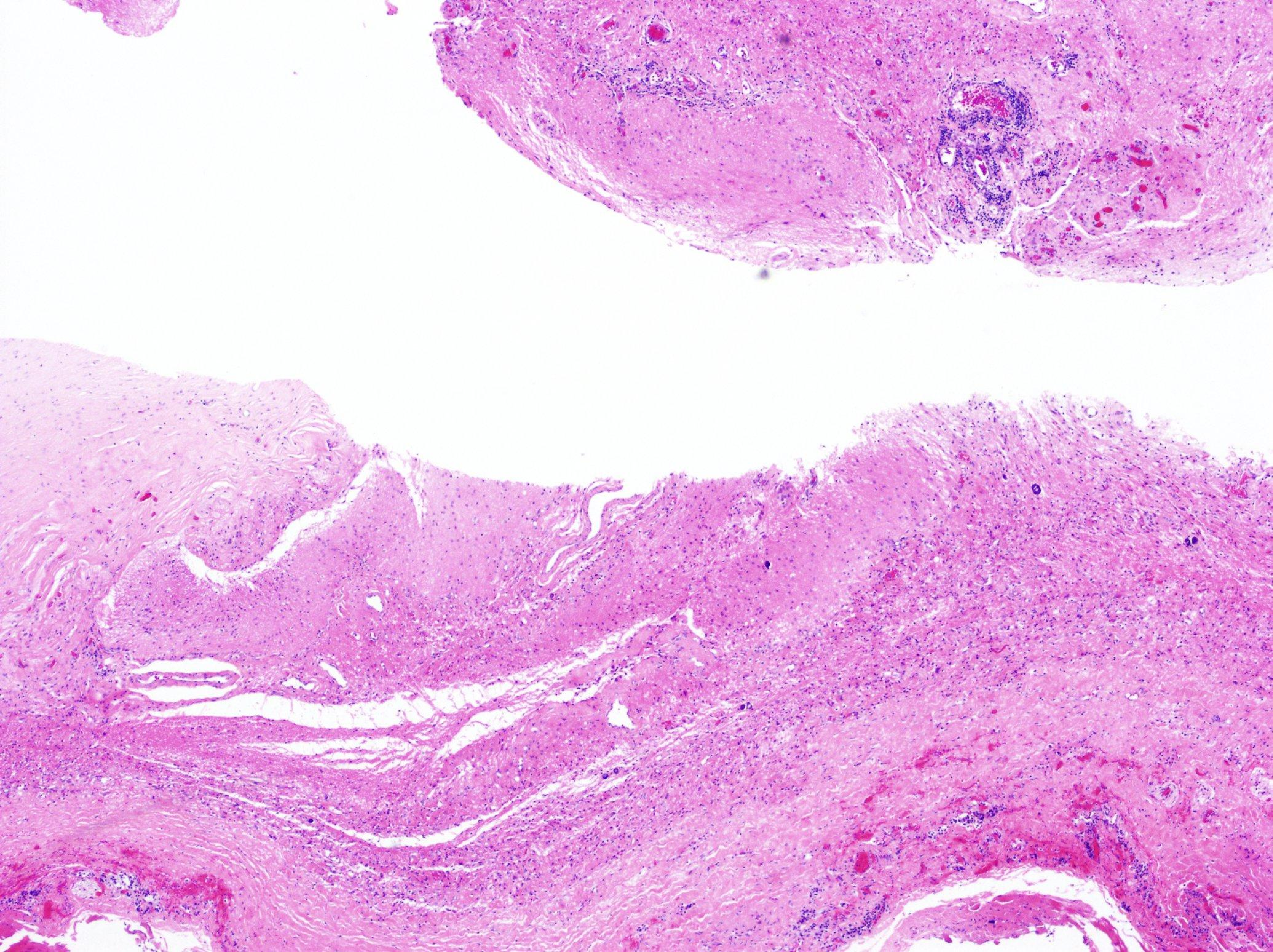

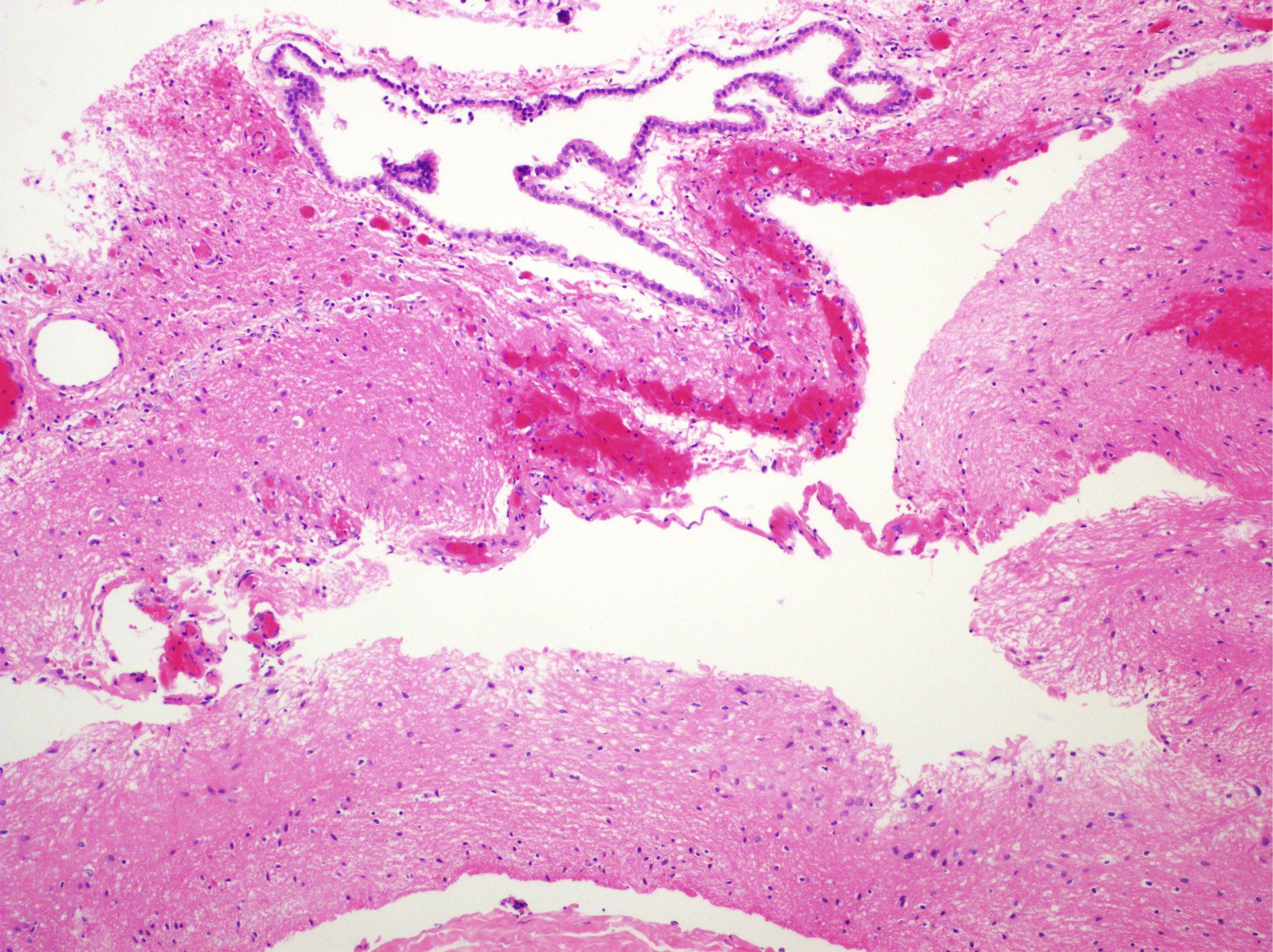

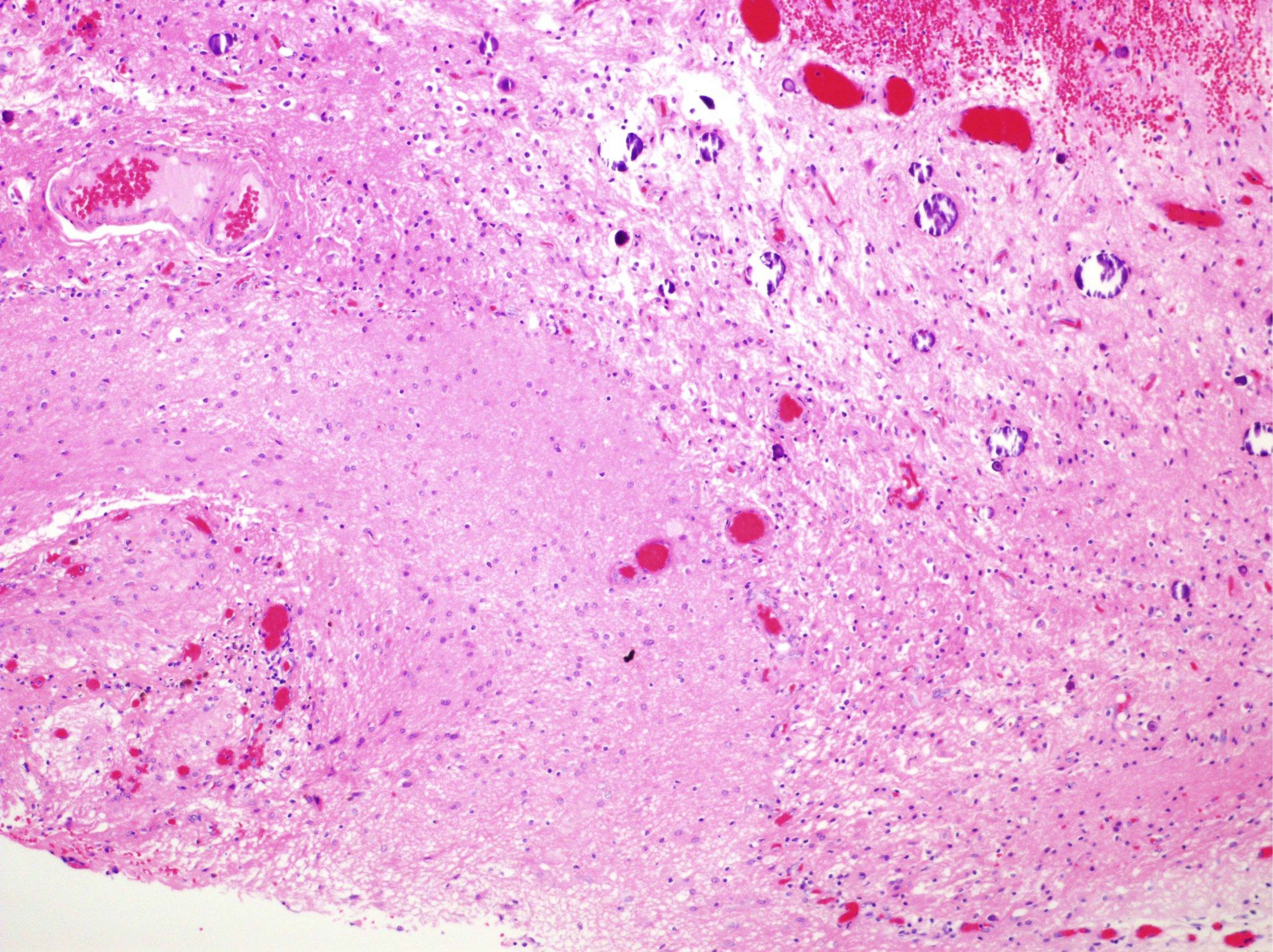

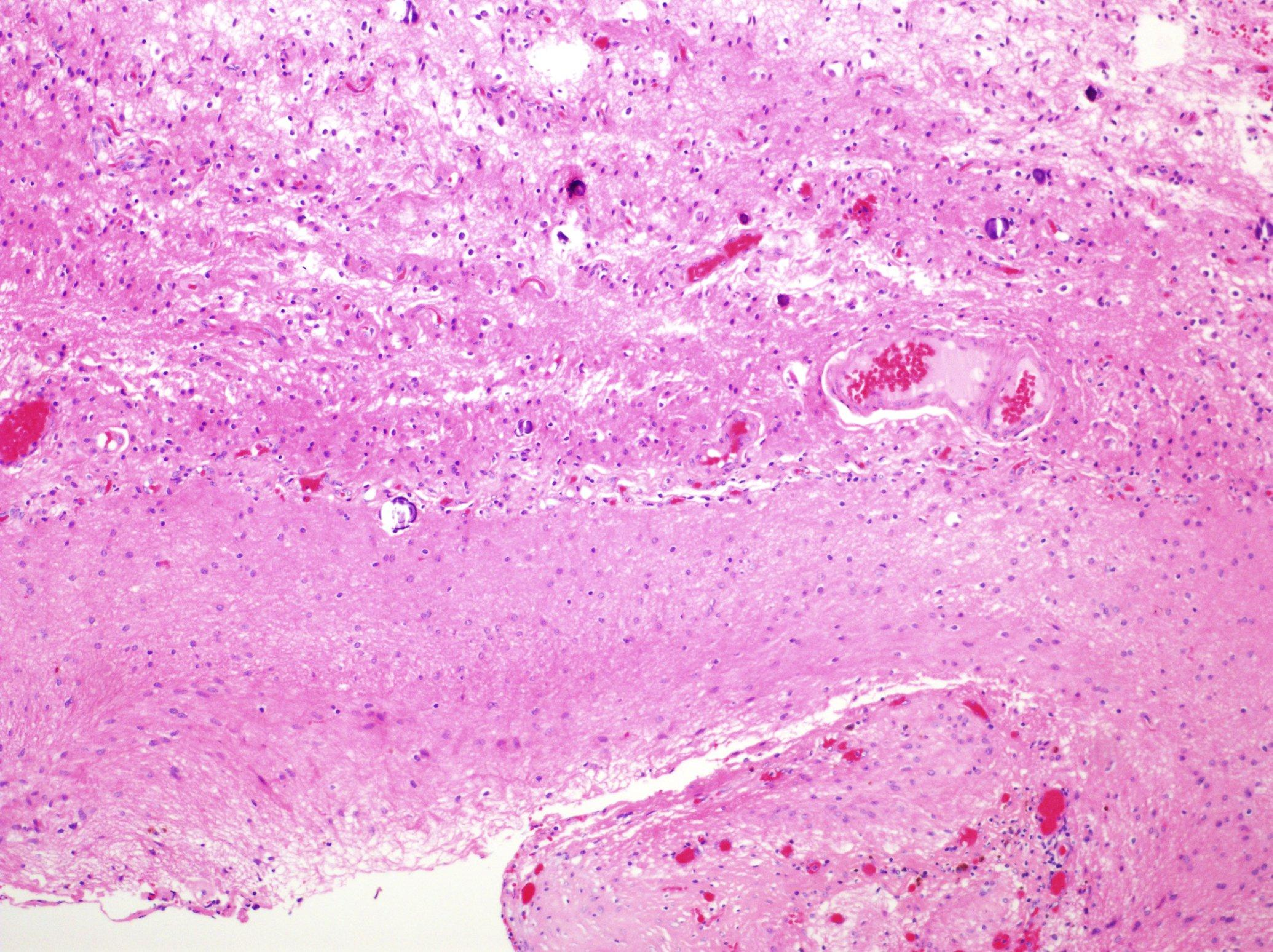

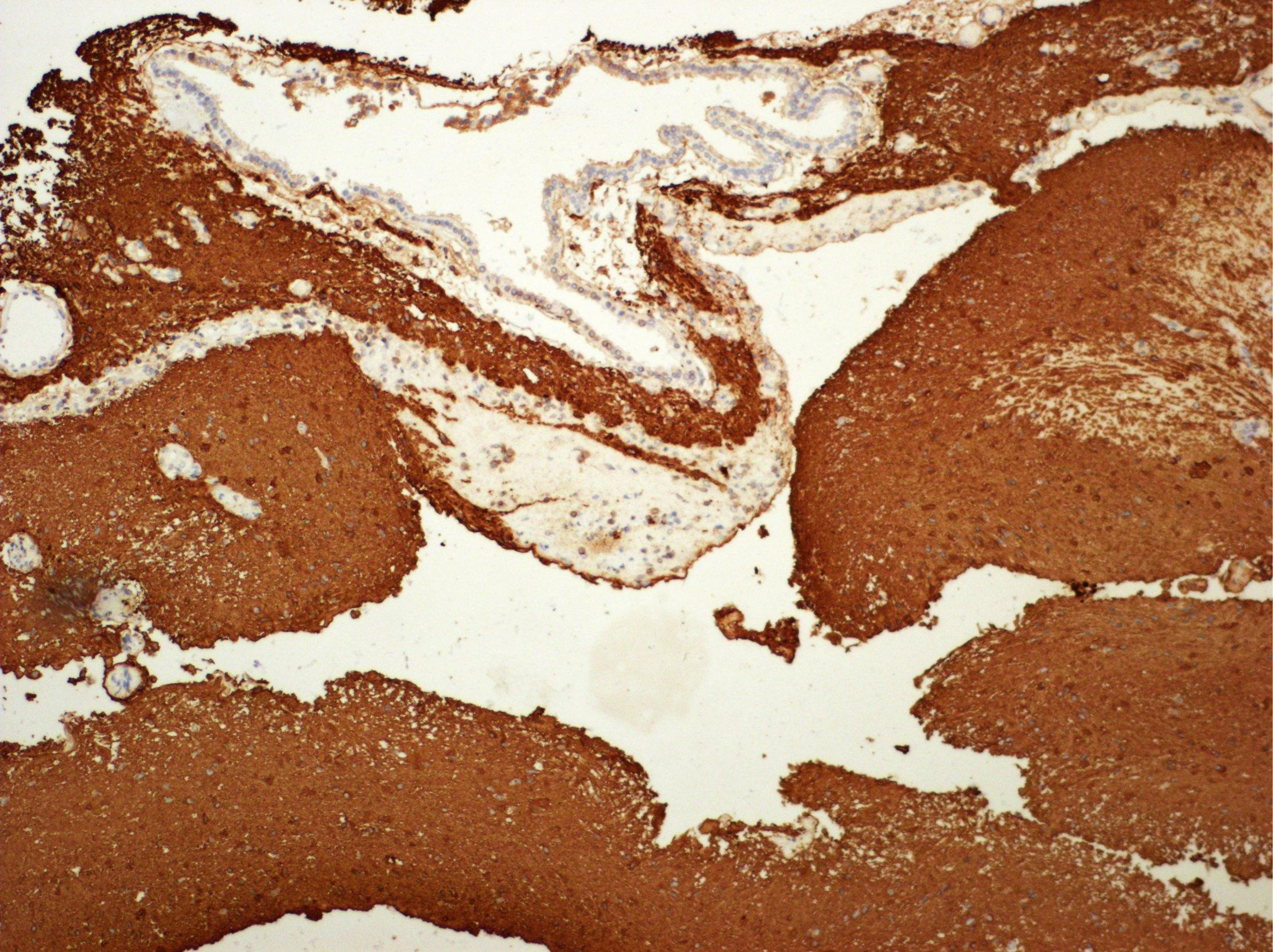

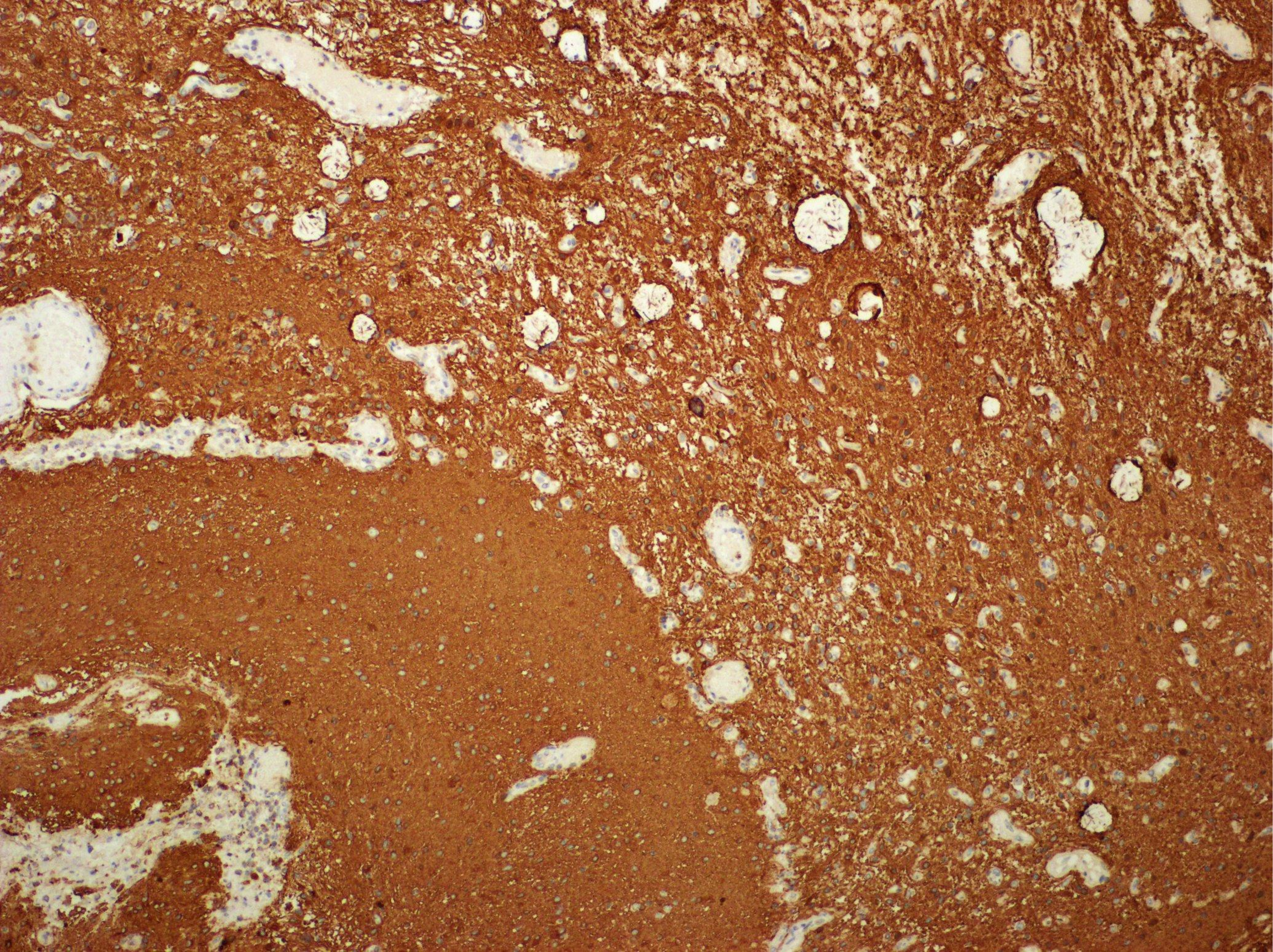

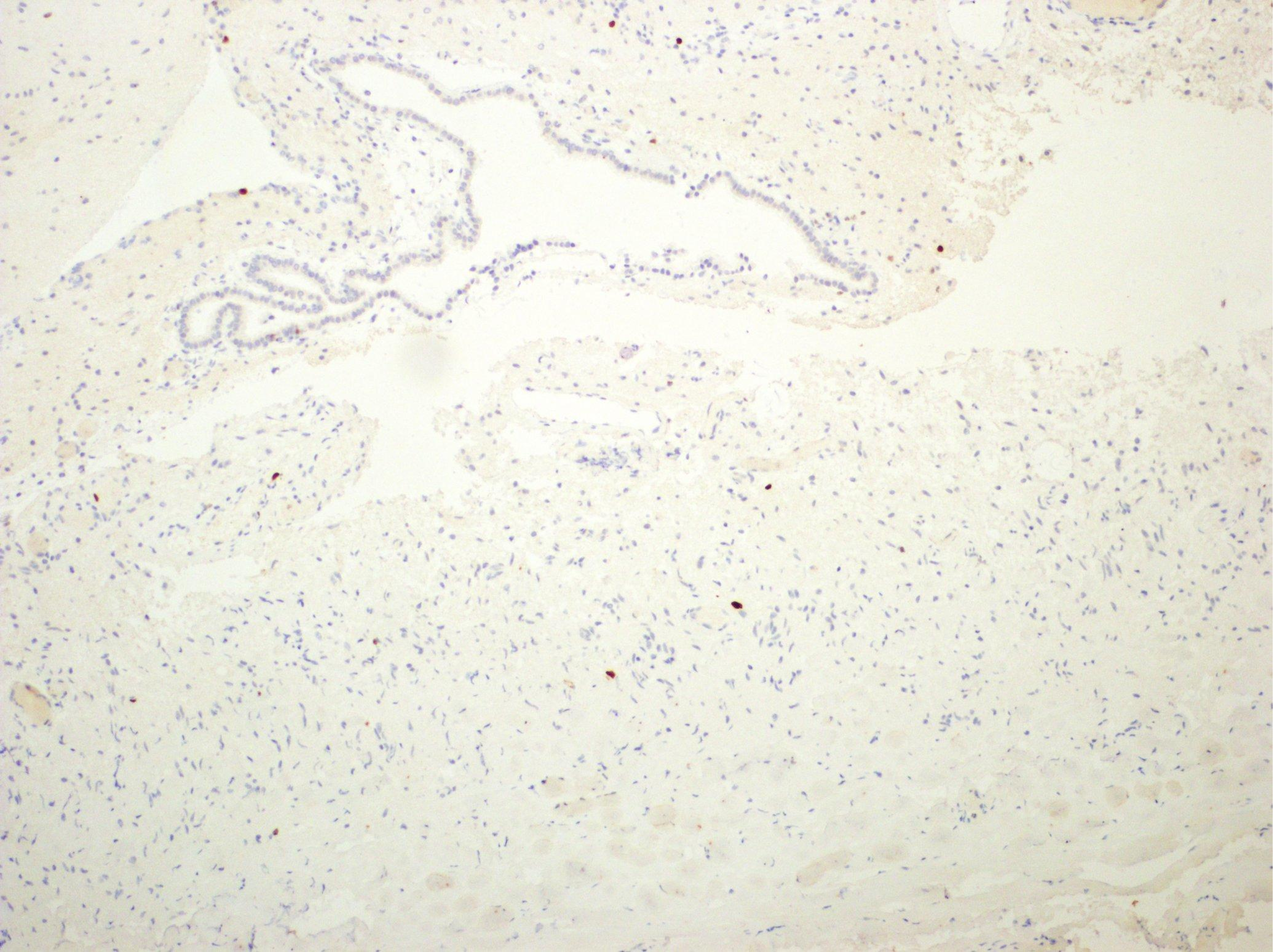

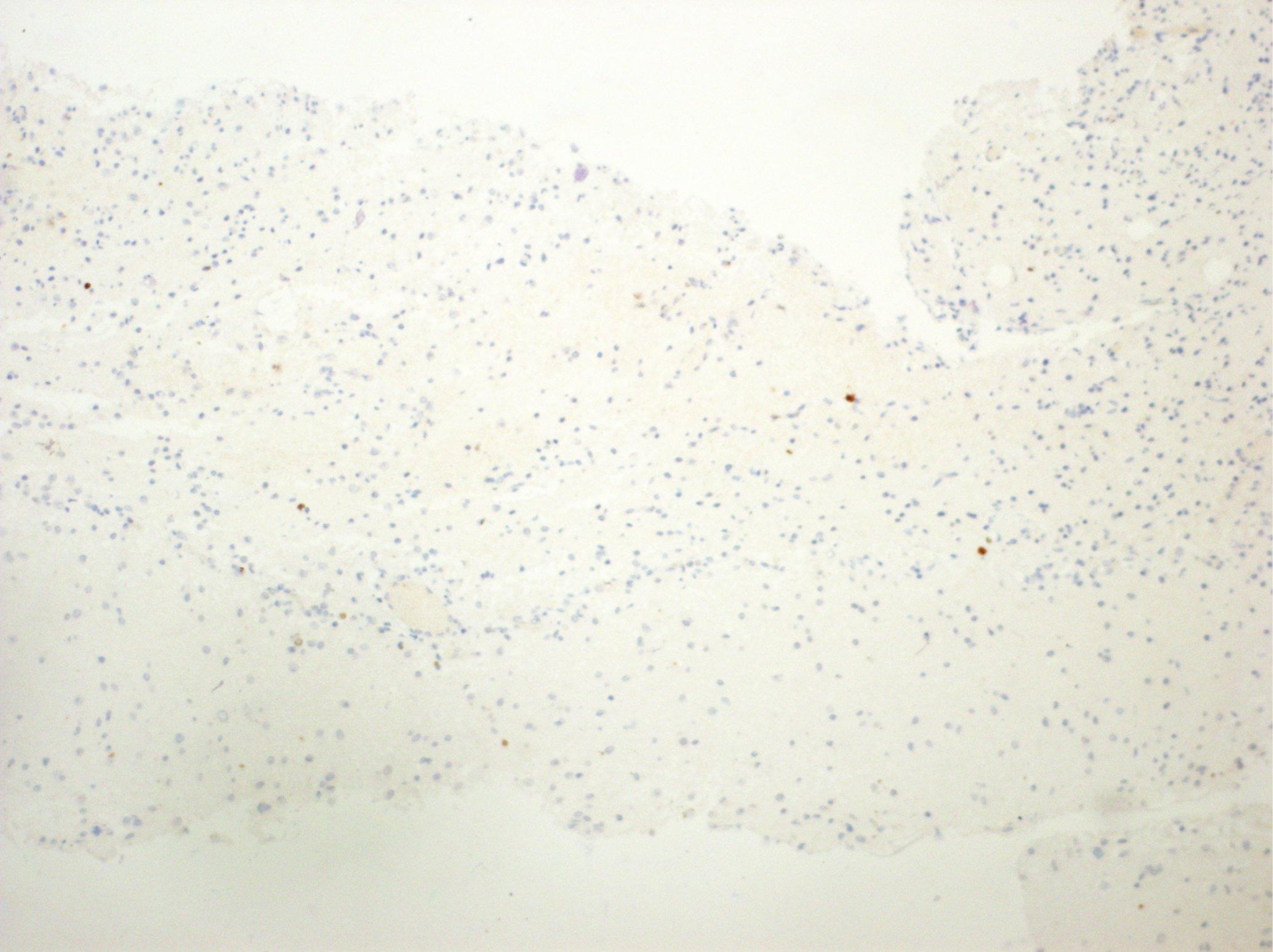

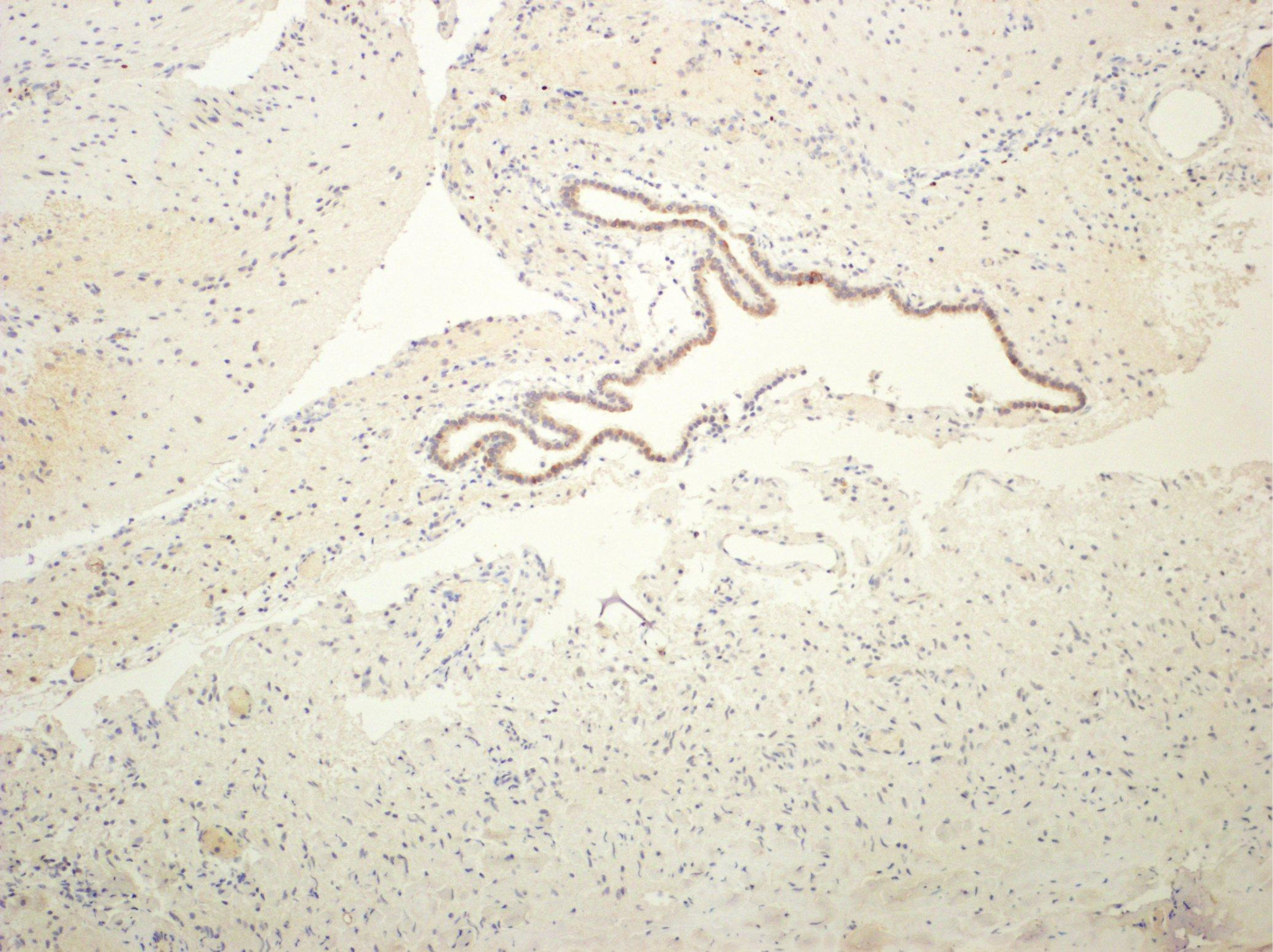

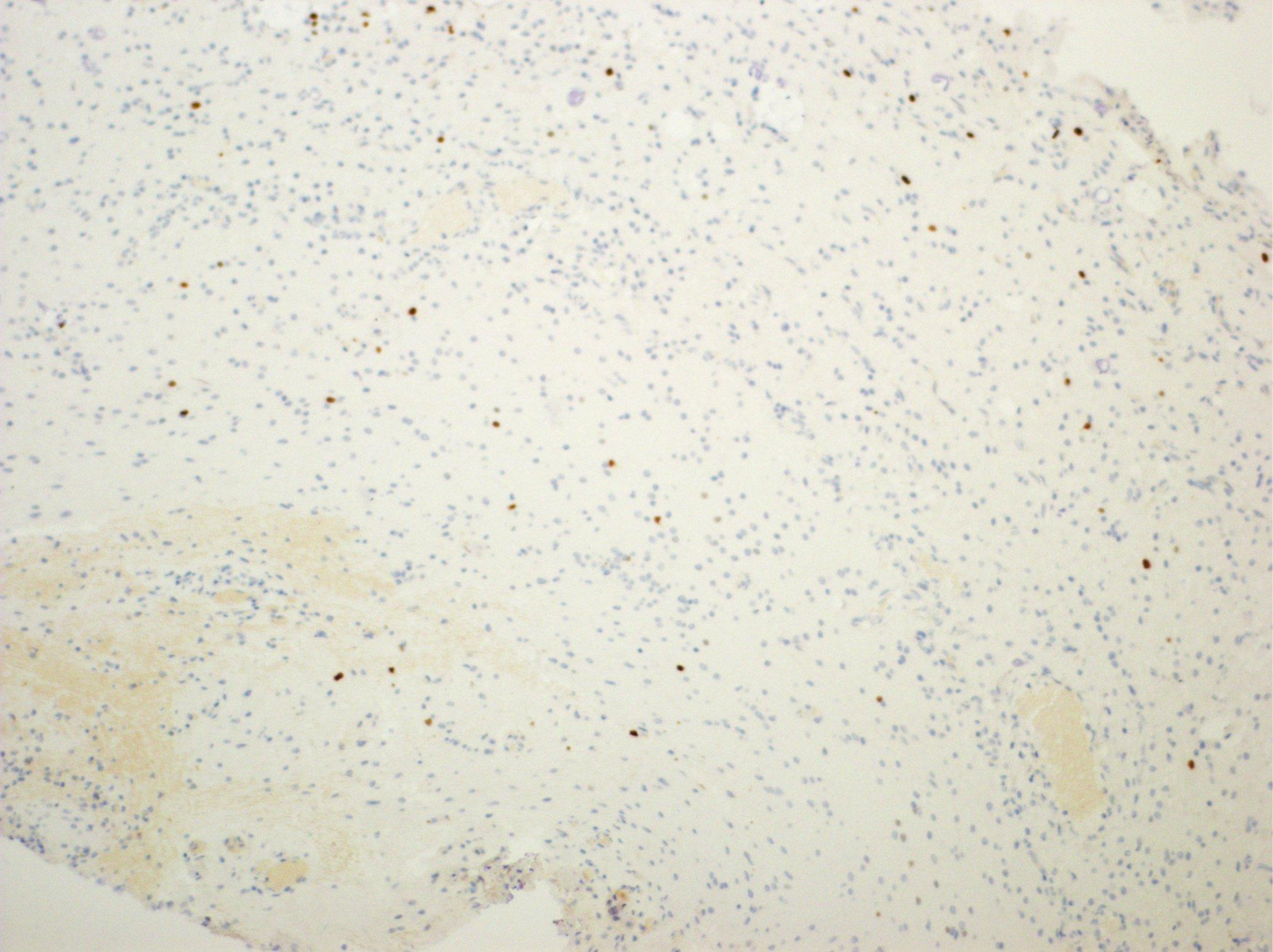

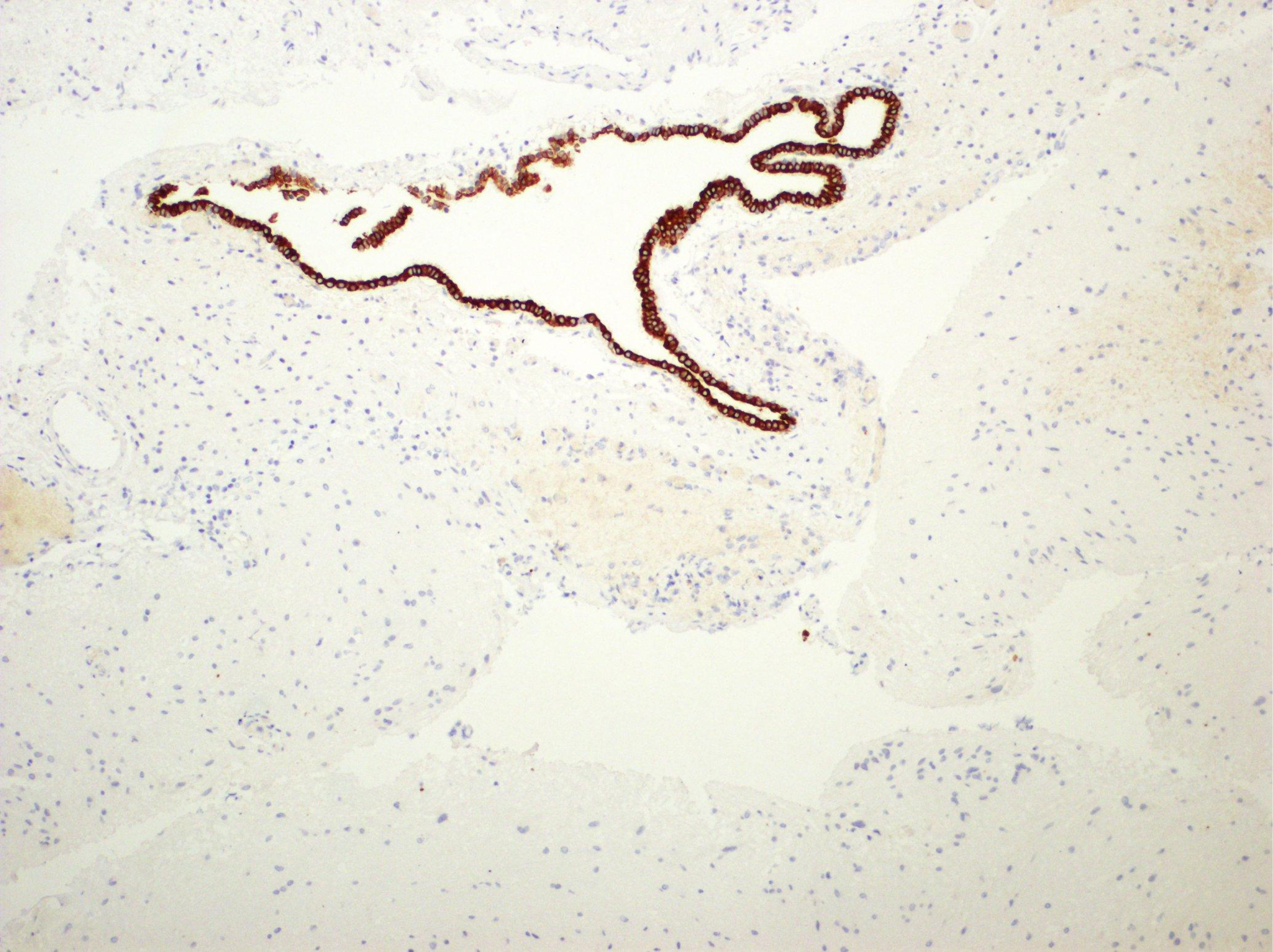

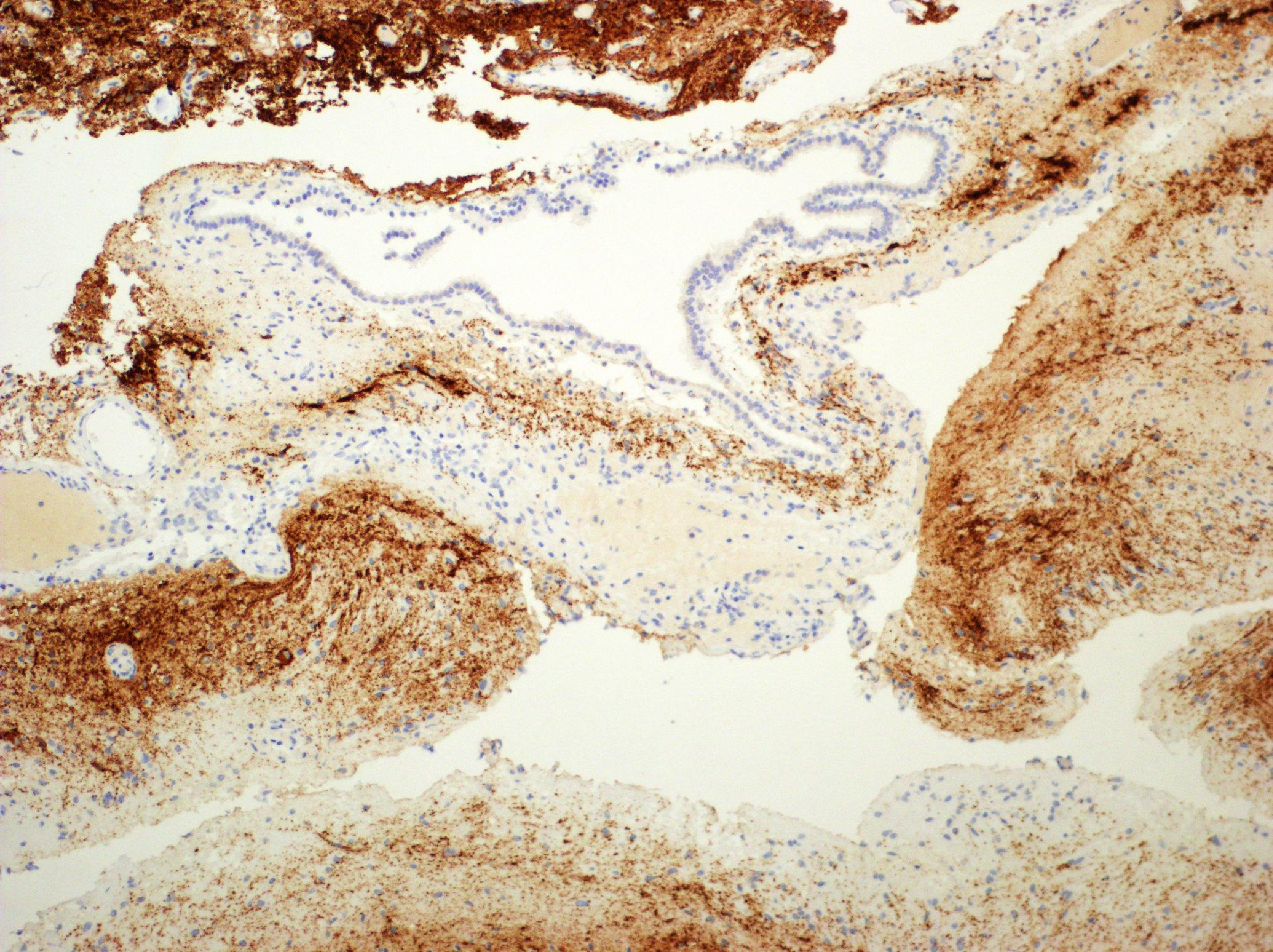

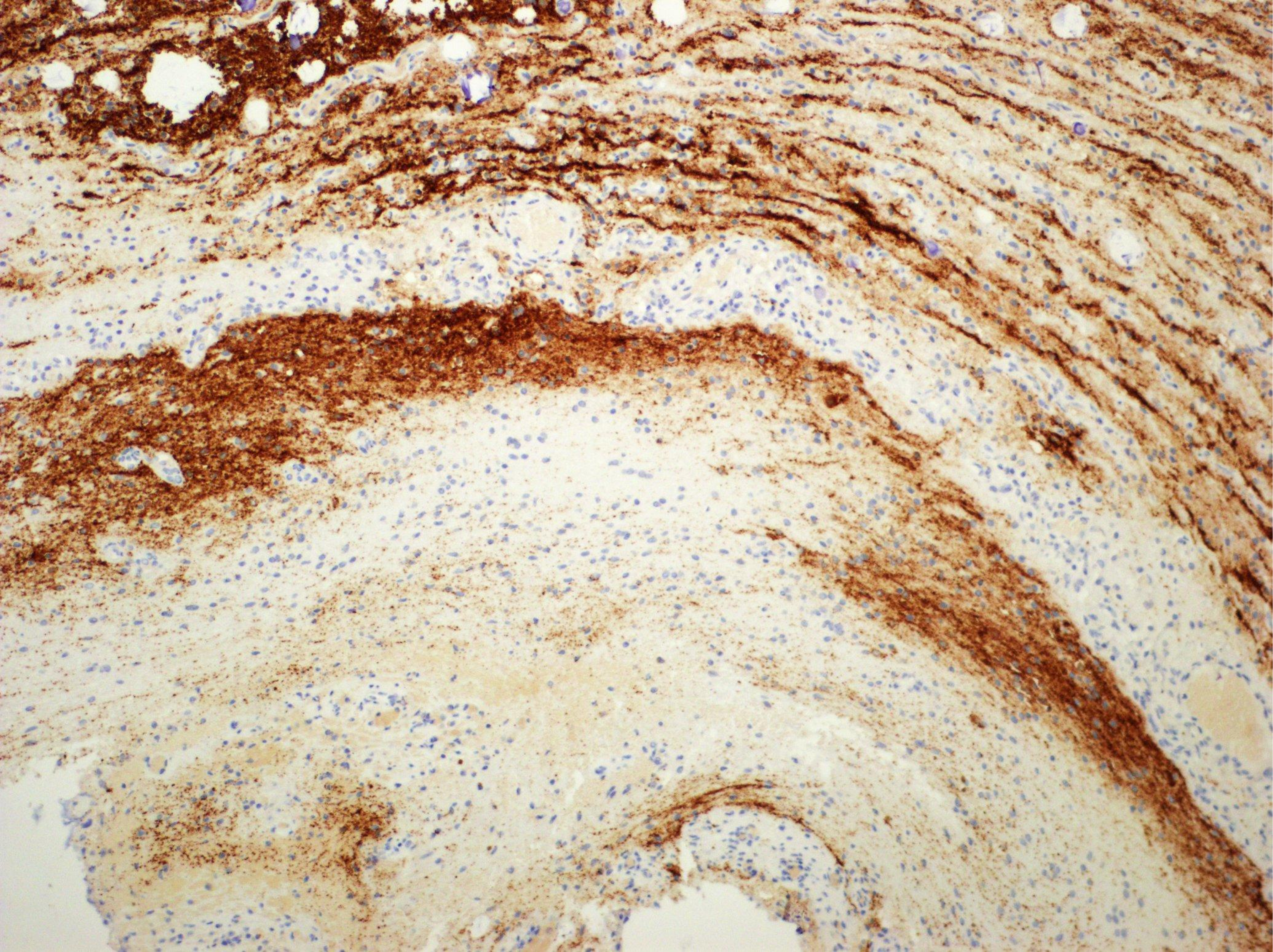

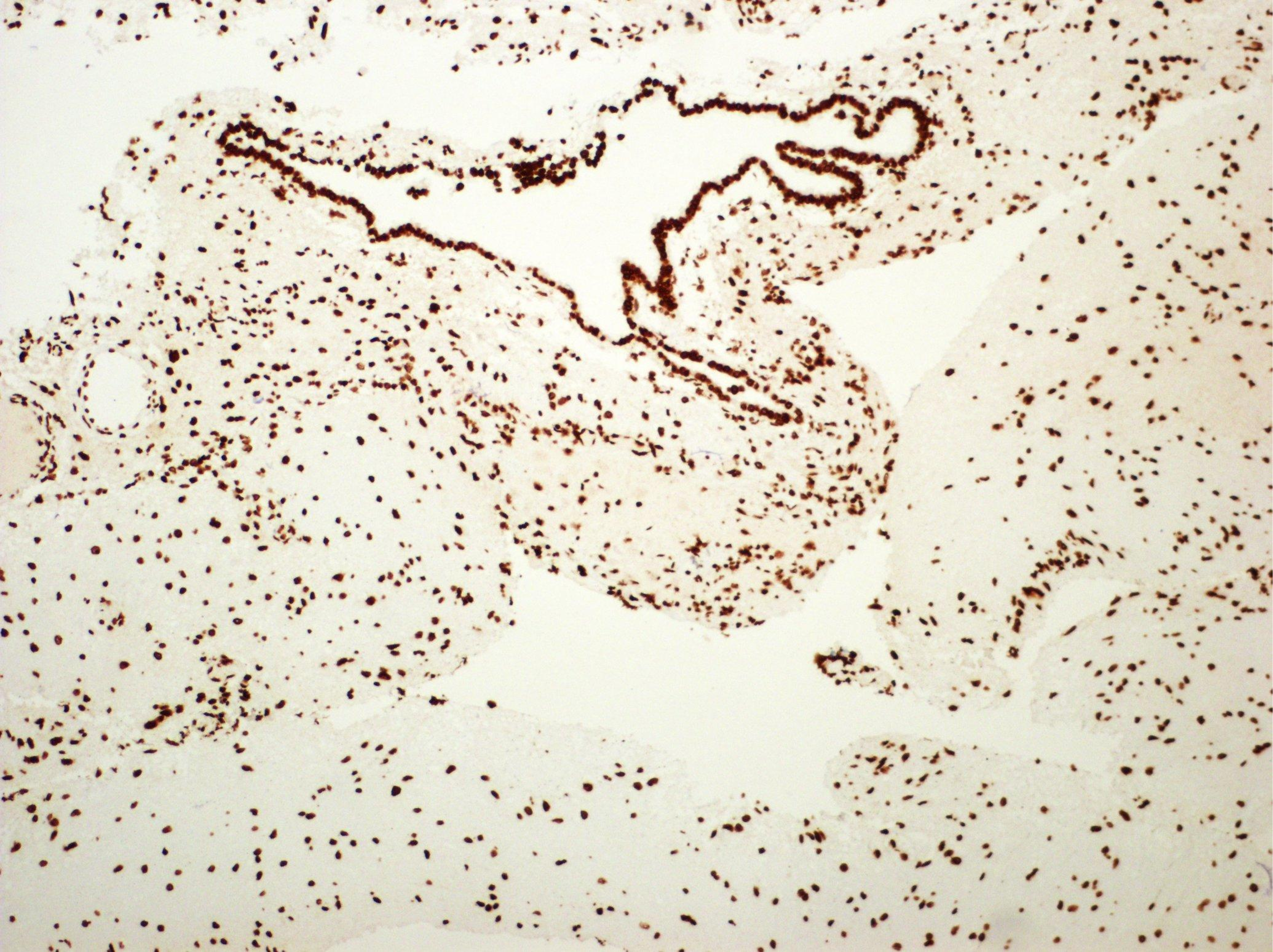

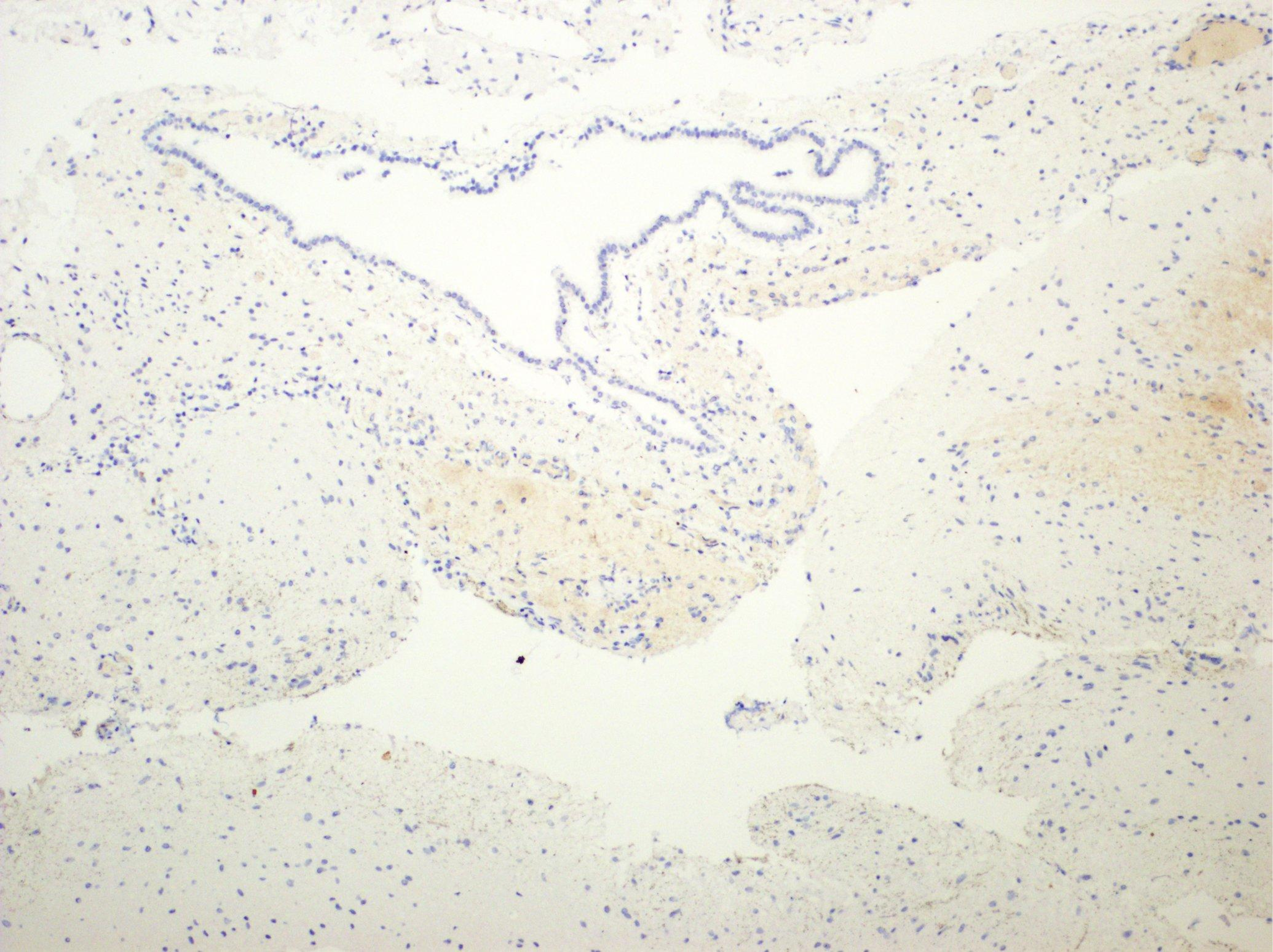

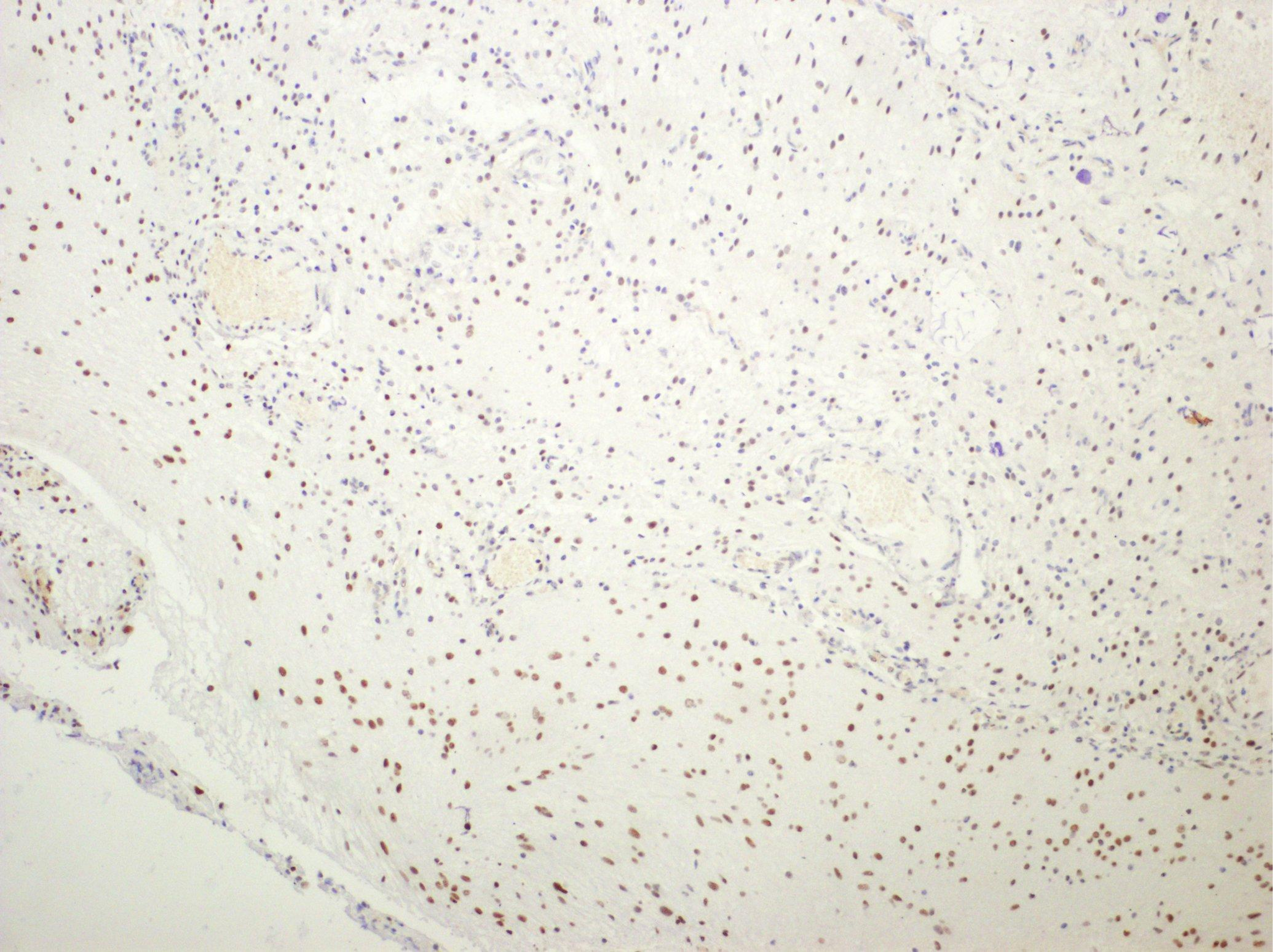

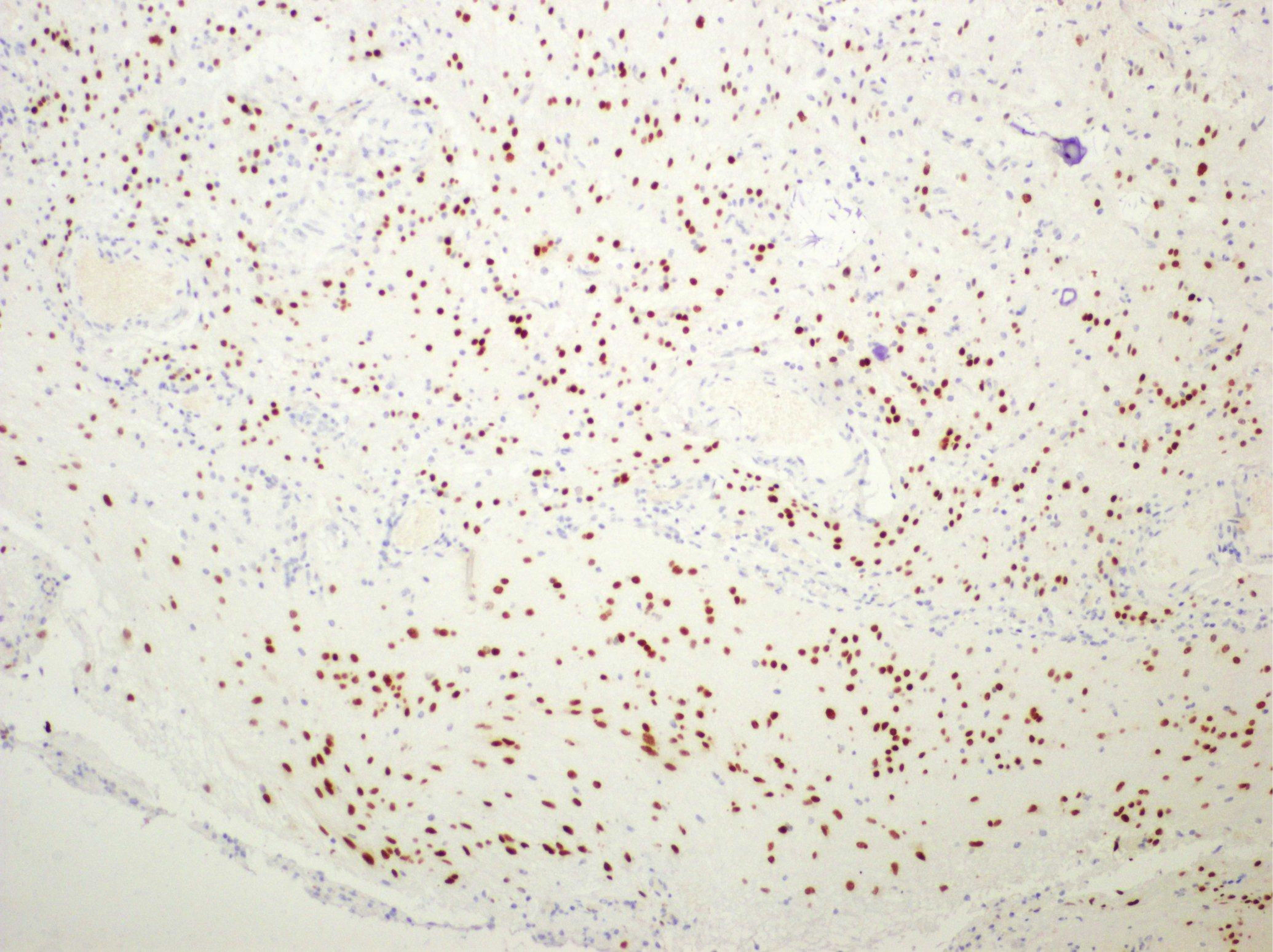

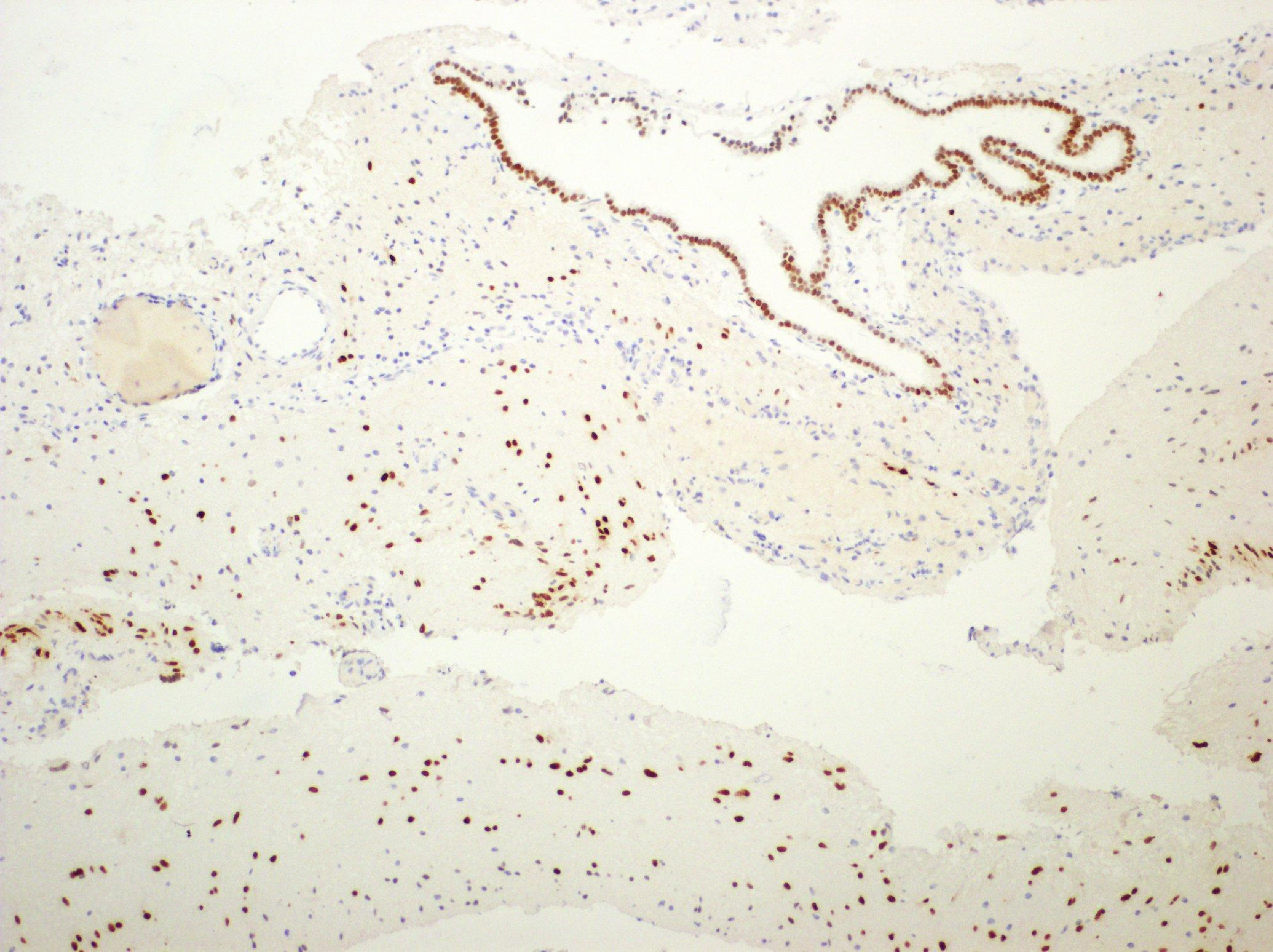

Supplement: Supplementary file 1 — Additional file 1. [file 12887_2023_4478_MOESM1_ESM.pdf]
